# Six Spain *Thymus* essential oils composition analysis and their in vitro and in silico study against *Streptococcus mutans*

**DOI:** 10.1186/s12906-023-03928-7

**Published:** 2023-04-05

**Authors:** Su-Yeon Park, Rifat Nowshin Raka, Xiu-Li Hui, Yang Song, Jin-Long Sun, Jie Xiang, Juan Wang, Jian-Ming Jin, Xu-Kai Li, Jun-Song Xiao, Hua Wu

**Affiliations:** 1 College of Chemical and Materials Engineering, Beijing Technology and Business University, Building No.1, Fucheng Road 11#, Haidian District, Beijing, 100048 China; 2 Department of Stomatology, General Hospital, Beijing, China; 3 Shanxi Key Laboratory of Minor Crop Germplasm Innovation and Molecular Breeding, College of Life Sciences, Shanxi Agricultural University, Taigu, China

**Keywords:** *Thymus* essential oil, Gas chromatography-mass spectrometry, *Streptococcus mutans*, Virulence factor, Molecular docking

## Abstract

**Background:**

*Streptococcus mutans* is a well-known oral pathogen that plays a critical role in the development of dental caries. Many studies have been directed to discover the chemical compounds present in natural products to inhibit the growth and biofilm formation activity of *S. mutans*. *Thymus* essential oils exhibit good inhibition on the growth and pathogenesis of *S. mutans*. However, details about the active compounds in *Thymus* essential oil and the inhibition mechanism still remain unclear. The aim of this study was to investigate the antimicrobial activity of 6 *Thymus* species (Three samples of *Thymus vulgaris*, two samples of *Thymus zygis*, and one sample of *Thymus satureioides* essential oils) on *S. mutans*, to identify the potential active components, and to reveal the underlying mechanism.

**Methods:**

The composition of *Thymus* essential oils was analyzed by gas chromatography-mass spectrometry. And its antibacterial effect was evaluated based on the bacterial growth, acid production, biofilm formation and genetic expression of virulence factors by *S. mutans*. Potential active components of the *Thymus* essential oil were identified using molecular docking and correlation analysis.

**Results:**

GC–MS analysis showed that the major components in the 6 Spain *Thymus* essential oils were linalool, *α*-terpineol, *p*-cymene, thymol and carvacrol. MIC and MBC analysis showed that 3 *Thymus* essential oils showed very sensitive antimicrobial activity, and were chosen for further analysis. The 3 *Thymus* essential oil exhibited a significant inhibitory effect on acid production, adherence and biofilm formation of *S. mutans* and the expression of virulence genes, such as *brpA*, *gbpB*, *gtfB*, *gtfC*, *gtfD*, *vicR*, *spaP* and *relA*. Correlation analysis showed that phenolic components, such as carvacrol and thymol, were positively related to DIZ value, which suggests that they are the potential antimicrobial components. Molecular docking between the *Thymus* essential oil components and virulence proteins also found that carvacrol and thymol exhibited strong binding affinity with functional domains of virulence genes.

**Conclusions:**

*Thymus* essential oil showed significant inhibition against the growth and pathogenesis of *S. mutans* depending on their composition and concentration*.* And phenolic compounds, such as carvacrol and thymol, are the major active components. *Thymus* essential oil could be used in oral healthcare products as a potential anti-caries ingredient.

**Supplementary Information:**

The online version contains supplementary material available at 10.1186/s12906-023-03928-7.

## Background

Dental caries is the most widespread and noncommunicable disease (NCD), and one of the main reasons for the hospitalization of children in some high-income countries [1]. It may cause irreparable destruction to the tooth enamel and cause difficulty with food intake as well as great distress if left untreated [2]. Although dental caries is less prevalent than it was in previous decades due to better oral hygiene of the global population and the addition of fluoride compounds into most toothpaste formulations. However, incidences of dental caries and oral diseases persist due to the increase in the availability of sugary foods, the change in diets and longer life expectancies [3].

The occurrence of dental caries is mainly associated with oral microbial pathogens, especially *Streptococcus mutans* (*S. mutans*) [4]. Dietary carbohydrates, especially sucrose could accelerate *S. mutans* cell propagation, cellular aggregation, biofilm formation and promote the film adherence by hydrophobic bonds to the enamel surface. Meanwhile, the sucrose metabolism subsequently leads the inner film's local acids secretion and accumulation to cause tooth enamel dissolution, decalcification, cavitation, and breakdown of the calcified dental tissue finally [5]. To form biofilm, produce acids and adhere to the enamel surface are recognized as virulence factors for dental caries [6].

As an obligate human pathogen in dental caries, the ability of *S. mutans* to assemble the insoluble exopolysaccharide (EPS) to form biofilms is marked as one of the most important dental virulence properties [7]. The EPS acts as a basal framework for the oral biofilm structure. Lots of studies indicated the biofilm cells exhibited over 1000-fold tolerant of antibiotics than planktonic cells [8]. Some genes and relative protein expression presented significant different profiles when the planktonic cells transform to the biofilm cells. The molecules commonly could help trigger and regulate the virulence factors, such as Gtfs, *vicR*, *gbpB*, *relA* and *spaP* etc. Gtfs can catalyze sucrose to synthesize EPS and promote the adhesion of *S. mutans* to tooth surfaces mainly [9]. *vicR* encodes putative histidine kinase, which regulates expression of *gbpB*, *gtfB*, *gtfC*, *gtfD* [10]. *gbpB* and *spaP* is also a factor that affects cell adhesion. *gbpB* mediates the interaction between the cell surface and glucan, while *spaP* plays a role in saliva-mediated aggregation and initial attachment to tooth surfaces. *brpA* and *relA* plays a critical role in the capacity of *S. mutans* to form stable biofilms and tolerate acid stress [11]. To control *S. mutans* cell propagation speed, downregulate the above genes’ expression and inhibit the biofilm cell transformation might be the useful ways to prevent dental caries [12].

Antibiotics and synthetic chemicals, such as fluoride, ampicillin, penicillin, and chlorhexidine were used as traditional antibacterial agents to prevent dental caries [13]. However, lots of cases indicated that *S. mutans* were easy to develop drug resistance to a single antibiotic after a long time or high-frequency use. Also, several adverse effects were found, such as teeth discoloration, taste alterations, mouth dryness, supragingival calculus accumulation, oral mucosal lesions tooth staining, vomiting, and even oral cancer [14]. Meanwhile, Essential oils, one kind of aromatic, organic, and small molecular natural metabolism products have been proven with a wide range of biological and pharmacological activities through numerous studies including dental diseases [15]. As alternative or combination antibacterial agents with minimal side effects and maximum antimicrobial effects, Essential oils (EOs) might be a good choice for caries prevention and oral problems [16].

The genus *Thymus* is aromatic perennial herb, which belongs to the Lamiaceae family, and it is native to temperate regions in Europe, North Africa, and Asia [17]. For centuries, Leaves and flowering parts of *Thymus* species are widely used as flavoring agents, culinary herb, and herbal medicine as well [18]. In Spain, thyme leaf and their extracts including essential oils commercially used in the food industry, especially as flavoring agents added to meat and fish [19]. *Thymus vulgaris* is widely used as folk medicine in ancient Europe for treatment of wounds, gastroenteric and bronchopulmonary disorders, due to its anthelmintic, expectorant, sedative diaphoretic, healing and antiseptic properties [20]. *Thymus zygis* grows in the countries around the Mediterranean Sea and is widespread throughout Portugal and Spain [21], and it is locally used as antiseptic and condiment in Portugal [22]. In Morocco, *Thymus satureioides*, one of the most popular herbs, is used in the cosmetic and perfume industries, and also for the food preservation [23].

Indeed, *Thymus* essential oil (TEO) is among the world's ten most used EOs as a food preservative [24]. Numerous studies indicated that TEO as well as its main volatile components thymol and carvacrol exert excellent bioactivities such as antibacterial, antiviral, antispasmodic, sedative, anti-inflammation and antioxidant [25]. Meanwhile, the studies revealed that the TEOs bioactivities were mainly determined by the composition and relative component’s content (metabolic features). TEO compounds contains various chemical groups including monoterpenes, monoterpene alcohols, phenol derivatives, ketones, aldehydes, ethers, and esters [17]. The TEOs’ metabolic features differ greatly according to germplasm (species/cultivar), regions, climate, cultural methods, extraction methods, and so on [26, 27].

To the best of our knowledge, the effects of *Thymus* species on antimicrobial activities have been characterized and reported, however, there are few studies on comparison of chemical compositions and antimicrobial mechanisms of several species of TEOs against *S. mutans*. In this study, we aim to detail the chemical constituents of six TEOs from three different species in Spain, as well as to investigate and compare their antimicrobial on the growth, acid-production, hydrophobicity, and biofilm formation of *S. mutans*. We also evaluated its influence on the expression of several virulence factors associated with bacterial adhesion and biofilm formation. The components from six TEOs were analyzed for bioactivity against the of virulence proteins.

## Methods

### Essential oils and bacterial strain

The six TEOs (Table 1) were obtained and their full botanical plant names were checked with the web (http://www.theplantlist.org) with help from the manager of Poli Aromatic Pharmaceutical Technology Co., Ltd in Shanghai, China. All the plants were grown in the same garden in Andalusia (Spain). TEOs were extracted by using hydro-distillation method locally in July of 2020. When we got them, they were dried using anhydrous sodium sulfate and stored at 4℃ until use.

**Table 1:** Collection site and chemical type of the six studied *Thymus* species from Spain

Code	Species	Chemical Type
TEO1	***Thymus zygis* L.**	Linalool
TEO2	***Thymus satureioides* Coss.**	Borneol
TEO3	***Thymus vulgaris* L.**	Linalool
TEO4	***Thymus vulgaris* L.**	Carvacrol
TEO5	***Thymus zygis* L.**	Thymol
TEO6	***Thymus vulgaris* L.**	Thymol

*Streptococcus mutans* (ATCC 700,610) was commercially obtained from the Microbial Species Conservation Center, Chinese Academy of Sciences. *S. mutans* was routinely grown in the Brain Heart Infusion (BHI) broth (pH 7.2, OXOID) at 37 °C under anaerobic conditions (85% N_2_, 5% CO_2_ and 10% H_2_).

### Gas chromatography/mass spectrometry (GC–MS) analysis

GC–MS analyses were performed using Agilent 6890 gas chromatograph equipped with an Agilent 5973 mass selective detector (Agilent Technologies, Folsom, CA), 60 m × 0.25 mm i.d., 0.25 µm film thickness, Agilent, USA. 1 μL of TEOs and 10 μL of 2—nonyl ketone was dissolved with 989 methylene chloride, mixed well, and filtered with a micro syringe. The temperature program was 50 °C for 3 min, then increased to 230 °C at 6 °C/min and held for 5 min. Helium at 1.3 mL/min constant flow was used as carrier gas under a splitless mode, and the injector was maintained at a temperature of 250 °C. The MS conditions were under 70 eV ionization energy, 100 °C quadrupole temperature, 1.4 scan/s scanning velocity, 45–350 amu weight range [28]. Compounds were calculated separately relative to NIST 17 Mass Spectral Library [29].

### Antimicrobial assay

The antimicrobial activities of six TEOs were screened by the standard disk diffusion susceptibility test on BHI solid media [30]. Briefly, the single colony of *S. mutans* was cultured in a 5 mL BHI liquid culture media for 15 h, and then the bacteria cell suspension was adjusted to a cell density of 10^5^ CFU/mL. Then 0.1 mL of this suspension was spread on the BHI agar culture media. One 3-mm-thick and 6-mm-diameter sterile filter paper discs were individually impregnated with 10 μL of each pure TEOs and were placed on the inoculated plates, and incubated at 37 °C for 24 h. A disc containing same dose of 1% penicillin–streptomycin (P/S) was placed in the plate as a positive control. The diameters of inhibition zones (DIZ) around the filter paper were measured in millimeters; the average and standard deviations were calculated to classify the TEOs as follows: *S. mutans* is not sensitive (0) for a DIZ than 8 mm, moderately sensitive ( +) for 8—20 mm DIZ, sensitive (+ +) for 20—30 mm DIZ, and very sensitive (+ + +) for DIZ more than 30 mm [31].

### Determination of minimum inhibitory and minimum bactericide concentration

TEOs that previously showed very sensitive antimicrobial activity (> 30 mm zone of inhibition) were screened for determination of MIC and MBC against *S. mutans* by the two-fold serial dilutions method with some modifications [32]. *S. mutans* cell suspension was obtained from the single colony cultured in a 5 mL BHI liquid culture media for 15 h, and adjusted to 2 × 10^8^ CFU/mL with sterile BHI liquid culture media. Two-fold dilutions of TEOs emulsified with Tween 80 (< 0.1% v/v at last) in BHI liquid media were prepared. 2 mL of aliquots of each TEOs’ dilutions were dispended in the tubes with 2 mL 2 × 10^8^ CFU/mL *S. mutans* cell suspension. *S. mutans* cell suspension containing 0.1% Tween 80 and 1% penicillin–streptomycin (P/S) were used as negative and positive controls, respectively. All the cultures were shaking at around 180 rpm/min for 24 h culture. MIC was determined as the highest dilution (lowest concentration) of the EO inhibiting visible bacterial growth. In order to confirm MBC, a 0.1 mL of the suspensions from the tubes showing no turbidity (i.e., MIC) was subcultured on BHI agar plates. MBC was determined as the highest dilution (lowest concentration) at which no growth occurred on the plates. The evaluation of MIC and MBC were carried out at least in triplicate. To determine the nature of antibacterial effect of TEOs, the MBC:MIC ratio for bacteria was used. When MBC: MIC ratio for *S. mutans* was 2: 1, the TEOs were considered bactericidal against *S. mutans*, and when the ratio was higher than 2: 1, it was considered bacteriostatic [33].

### Determination of *S. mutans* acid production

The inhibition effects of TEOs around active concentrations against the acid production of *S. mutans* were evaluated by the broth dilution method. Briefly, different concentrations of each TEO were diluted as aforementioned methods, then added to the same volume of culture media contained with 2 × 10^5^ CFU/mL *S. mutans* cell suspension. The liquid culture media used in this study contained an extra 1% (m/v) glucose. The pH value of each treatment was directly measured in the bacteria growth media by the pH meter (Mettler-Toledo International Inc, MTD, Shanghai), after 24 h cultivation [34]. All the treatments were tested at least in triplicate.

### Determination of *S. mutans* hydrophobicity

Different concentrations of TEOs were added to 3 mL containing 1% sucrose at 10^5^ CFU/mL *S. mutans* suspension and incubated under anaerobic conditions at 37 °C. Bacterial cells from late-exponential-phase cultures were washed twice and suspended in PUM buffer (22.2 g of K_2_HPO_4_·3H_2_O, 7.26 g of KH_2_PO_4_, 1.8 g of urea, 0.2 g of MgSO_4_·7H_2_O, and distilled water to 1,000 mL, pH 7.1) to an OD_600nm_ of 0.5 ~ 0.6. Then, 0.4 mL of hexadecane (Sigma-Aldrich) was added to 3 mL of the cell suspensions. The mixtures were vortexed for 60 s, and the aqueous phase could settle for 15 min. The percentage of cells partitioned to hexadecane was calculated as (OD_600nm_ before adsorption × OD_600nm_ after adsorption) / (OD_600nm_ before adsorption) × 100 [34].

### Biofilm formation assay

Safranine staining and crystal violet staining were used to evaluate the biofilm formation. Various concentrations (1/8—2MIC) of TEOs were added to BHI broth containing 0.1% sucrose in 35 mm polystyrene dishes and 96-well plates (Corning, NY, USA). The cultures were inoculated with 1 × 10^5^ CFU/mL *S. mutans* and incubated under anaerobic conditions at 37 °C for 48 h. After incubation, the supernatants were removed and the culture dishes and plates were rinsed with distilled H_2_O twice. With 0.1% safranin staining, the biofilm formation features of dishes were measured by visually visualized and photographed [35]. Biofilm formation in the 96-well plates was stained with 0.1 mL 0.4% crystal violet for 15 min, and then dissolved in 95% ethanol. The optical densities of isolated ethanol solution were quantified at 540 nm for quantity analysis. The inhibitory rate of antibiofilm formation was calculated and demonstrated according to the equation: Inhibitory rate = (1 − S/C) × 100% (C and S were defined as the average absorbance of control and sample groups respectively) [36].

### Expression analysis of different *S. mutans* virulence genes

To determine the effects of TEOs on the virulence gene expression of *S. mutans*, RT-qPCR assay was performed [12]. The bacteria (approximately 1 × 10^5^ CFU/mL) with different concentrations TEOs treatment were cultured under anaerobic conditions at 37 °C for 24 h. Total RNA was extracted from the collected bacteria by using a bacterial total RNA kit (OMEGA, USA), and cDNA was synthesized by using a reverse transcription kit (Takara, JAPAN). A Thermal Cycler (ABI, USA) and SYBR Green detection dye (Applied Bio systems Inc) were used in the RT-qPCR amplification. The PCR condition included an initial denaturation at 95 °C for 2 min, followed by 40 cycles of denaturation at 95 °C for 30 s, annealing at 57 °C for 30 s and extension at 72 °C for 30 s. 16S rRNA was used as an internal control. 16SrRNA was selected as an internal standard and all primers for real-time PCR were designed with Primer5 according to the Genebank sequence of *S. mutans* UA159 (Table 2).

**Table 2:** Primers sequences for gene expression detection by qRT-PCR

Genes (Gene description)		Primer Sequence
*16 s rRNA*	Forward	CCTACGGGAGGCAGCAGTAG
	Reverse	CAACAGAGCTTTACGATCCGAAA
*gtfB*	Forward	AGCAATGCAGCCAATCTACAAAT
	Reverse	ACGAACTTTGCCGTT ATTGTCA
*gtfC*	Forward	GGTTTAACGTCAAAATTAGCTGTATTAGC
	Reverse	CTCAACCAACCGCCACTGTT
*gtfD*	Forward	ACAGCAGACAGCAGCCAAGA
	Reverse	ACTGGGTTTGCTGCGTTTG
*brpA*	Forward	GGAGGAGCTGCATCAGGATTC
	Reverse	AACTCCAGCACATCCAGCAAG
*gbpB*	Forward	ATGGCGGTTATGGACACGTT
	Reverse	TTTGGCCACCTTGAACACCT
*relA*	Forward	ACAAAAAGGGTATCGTCCGTACAT
	Reverse	AATCACGCTTGGTATTGCTAATTG
*spaP*	Forward	GACTTTGGTAATGGTTATGCATCAA
	Reverse	TTTGTATCAGCCGGATCAAGTG
*vicR*	Forward	TGACACGATTACAGCCTTTGATG
	Reverse	CGTCTAGTTCTGGTAACATTAAGTCCAATA

### Molecular docking

All the protein sequences were collected from uniprotkb. The protein structures were downloaded from Protein Data Bank (PDB) or designed using Swiss-Model and I-tasser by homology modeling. After the validation of the designed proteins, the most suitable 3D structures were used for docking. The most important 10 components for all 6 TEOs were selected as ligands and downloaded from the PubChem database. The ADMET analysis of those components was done by the SwissADME server. The docking was performed using CBdock and pockets were determined in the process (see supplementary data Figure S1). The functional domains of those proteins were analyzed and predicted by literature reviews and Interpro based on their sequences.

### Statistical analysis

All experiments were performed in triplicate. Duncan’s analysis of variance was performed by SPSS 19.0 (SPSS Incorporated, Chicago) after one-way classifications (ANOVA) investigation based on the parameters. All the data were presented as mean ± standard deviation. Values were considered statistically significant if *p* < 0.05. Mapping was performed using Origin8.5.1.

## Results

### Chemical composition

Through the GC–MS analysis, there were 52 components with a relative peak area percentage of more than 0.05% recorded and analyzed with 3 species and 6 cultivars’ TEOs from Spain (Table 3). The 52 compounds could be classified and summed as monoterpenoids (19.07–38.37%), sesquiterpenoids (1.57–8.68%), ketones (0–4.82%), alcohols (12.2–63.62%), esters (0–4.55%), phenols (0.58–48.82%), and others (0–4.26%). It was found that *α*-terpineol, carvacrol, linalool, *p*-cymene and thymol were the main chemicals in different TEOs separately.

**Table 3:** The main components of six different *Thymus* essential oils

No	Compounds	RI	Relative peak area (%)					
			**TEO 1**	**TEO 2**	**TEO 3**	**TEO 4**	**TEO 5**	**TEO 6**
***Monoterpenoids***								
1	*α*-Pinene	1028	1.81	2.79	2.01	1.24	0.25	0.68
2	*α*-Thujene	1029	0.06	-	-	0.66	-	0.22
3	Camphene	1071	1.12	8.13	0.86	0.19	1.71	2.06
4	*β*-Pinene	1112	0.38	0.89	0.39	0.36	0.26	0.3
5	Sabinene	1124	0.93	-	-	0.06	-	-
6	3-Carene	1147	-	-	-	0.21	0.08	0.11
7	*β*-Myrcene	1161	7.95	0.23	1.67	4.29	1.84	1.98
8	*α*-Phellandrene	1167	-	0.03	-	-	-	-
9	*α*-Terpinene	1180	3.64	0.51	-	2.24	1.65	1.73
10	Limonene	1200	3.37	1.35	1.26	0.62	0.64	0.79
11	*β*-Phellandrene	1211	1.35	-	-	0.8	-	-
12	*γ*-Terpinene	1246	6.36	1.42	-	7.6	10.86	9.9
13	*β*-Ocimene	1250	0.26	-	-	-	-	-
14	*p*-Cymene	1272	2.65	3.45	32.18	11.77	16.82	15.45
15	Terpinolene	1283	1.87	0.27	-	0.22	0.15	0.15
16	*p*-Cymenene	1444	-	-	-	0.12	0.1	-
17	trans-Sabinene hydrate	1460	0.69	-	-	0.81	0.23	0.31
***Sesquiterpenoids***								
18	*α*-Copaene	1492	-	0.26	-	-	-	-
19	*β*-Caryophyllene	1595	1.57	7.47	2.09	5.87	2.7	3.08
20	Alloaromadendrene	1635	-		-	-	0.08	-
21	*α*-Humulene	1667	-	-	-	0.21	-	-
22	*γ*-Gurjunene	1674	-	-	-	-	0.12	-
23	*γ*-Muurolene	1692	-	0.52	-	-	-	-
24	*δ*-Cadinene	1758	-		-	-	0.29	0.42
25	Caryophyllene oxide	1989	-	0.43	-	1.02	0.48	0.52
***Ketones***								
26	3-Octanone	1253	-	-	-	-	0.31	0.4
27	Camphor	1518	0.56	1.88	0.51	-	4.07	4.42
28	trans-Dihydrocarvone	1624	0.35	-	-	-	-	-
***Alcohols***								
29	3-Octanol	1393	-	-	-	0.06	0.14	0.15
30	Linalool oxide	1445	0.82		-		0.33	0.59
31	1-Octen-3-ol	1457	0.16	-	-	0.73	0.1	-
32	trans-Linalool oxide (furanoid)	1463	0.84	-	-	-	0.27	-
33	Linalool	1547	39.37	4.98	9.3	7.63	7.46	7.71
34	1-Terpinenol	1576	-	-	1.75	-	-	-
35	Terpinen-4-ol	1602	15.92	3.13	0.57	2.86	1.88	2.23
36	3,7-Dimethyl-1,5,7-octatrien-3-ol	1613	1.34	-	-	-	0.22	0.26
37	*β*-Terpineol	1627	-	-	1.7	-	-	-
38	*δ*-Terpineol	1682	-	-	-	-	0.09	-
39	*α*-Terpineol	1697	-	46.09	17.72	-	-	-
40	Borneol	1702	5.17	-	0.45	0.92	6.51	7
41	*p*-Cymen-8-ol	1852	-	-	-	-	0.36	0.53
42	*p*-Cymen-7-ol	2113	-	-	-	-	0.09	0.08
43	Spathulenol	2136	-	-	-	-	0.17	0.23
***Esters***								
44	Linalyl acetate	1555	-	-	-	-	0.1	-
45	Bornyl formate	1588	0.18	0.9	-	-	0.42	0.49
46	Bornyl acetate	1592	0.2	3.65	-	-	0.24	0.29
***Phenols***								
47	Thymyl methyl ether	1590	-	1.29	-	-	-	-
48	Eugenol	2139	-	-	-	0.17	-	-
49	Thymol	2189	0.58	3.22	-	3.99	30.48	27.96
50	Carvacrol	2236	-	5.35	26.41	44.66	4.85	5.35
***Others***								
51	Eucalyptol	1213	-	1.31	0.86	-	3.13	3.74
52	Carvacrol methyl ether	1601	-	-	-	-	0.39	0.52
**Monoterpenoids**			32.44	19.07	38.37	31.19	34.59	33.68
**Sesquiterpenoids**			1.57	8.68	2.09	7.1	3.67	4.02
**Ketones**			0.91	1.88	0.51	0	4.38	4.82
**Alcohols**			63.62	54.2	31.49	12.2	17.62	18.78
**Esters**			0.38	4.55	0	0	0.76	0.78
**Phenols**			0.58	9.86	26.41	48.82	35.33	33.31
**Others**			0	1.31	0.86	0	3.52	4.26
**Total (%)**			**99.50**	**99.55**	**99.73**	**99.31**	**99.87**	**99.65**

The samples were TEO1 (*T. zygis* CT linalool), TEO2 (*T. satureioides* CT borneol), TEO3 (*T. vulgaris* CT linalool), TEO4 (*T. vulgaris* CT carvacrol), TEO5 (*T. zygis* CT thymol) and TEO6 (*T. vulgaris* CT thymol). Identification method: compounds identified by comparison of mass spectra with NIST MS libraries after background correction

As shown in Table 3, two chemotypes of *T. zygis* were investigated, the “linalool” type (TEO1) with 39.37% linalool and 15.69% terpinen-4-ol as the main constituents, while the “thymol” type (TEO5) with 30.48% thymol and 4.85% carvacrol. The analysis 3 chemotypes of *T. vulgaris* showed that *p*-cymene (32.18%), carvacrol (26.41%), *α*-terpineol (17.72%), and linalool (9.3%) were the main constituents of “linalool” type (TEO3), “carvacrol” type (TEO4) contained 44.66% carvacrol, while the “thymol” (TEO6) contained 27.96% thymol and 5.35% carvacrol. *T. satureioides* (TEO2) contained a high amount of *α*-terpineol (46.09%).

### Screening of antibacterial activity

The DIZ screening of *S. mutans* with 6 TEOs presented different sensitives (Table 4). TEO6 gave the strongest inhibition activity with diameter values in the range of 39.3 ± 0.70 mm, followed by TEO4 and TEO5 with zones of inhibition 39.3 ± 0.70 mm and 33.4 ± 1.10 mm, respectively. The DIZ of TEO3 and TEO2 were smaller, at 29.5 ± 2.60 mm and 19.5 ± 1.00 mm, respectively. The lowest one was TEO1 with an 8.8 ± 0.20 mm zone. The TEOs with a DIZ of more than 30 mm were used in the following experiments.

**Table 4:** Antibacterial activity of different amounts of the *Thymus* essential oils

Essential oils	DIZ (mm)	Sensitivity index	MIC (μL/mL)	MBC (μL/mL)	MBC: MIC
TEO1	8.8 ± 0.20	+	-	-	-
TEO2	19.5 ± 1.00	+	-	-	-
TEO3	29.5 ± 2.60	+ +	-	-	-
TEO4	31.1 ± 0.20	+ + +	1.25	5.00	4:1
TEO5	33.4 ± 1.10	+ + +	0.625	1.25	2:1
TEO6	39.3 ± 0.70	+ + +	0.625	2.50	4:1
P/S	41.1 ± 2.20	+ + +	0.078125	0.625	8:1

Inhibition is expressed in mm and include the diameter of paper disc (6 mm). Results are shown as not sensitive (0) for a DIZ than 8 mm, moderately sensitive ( +) for 8—20 mm DIZ, sensitive (+ +) for 20—30 mm DIZ, and very sensitive (+ + +) for DIZ more than 30 mm; Minimal inhibitory concentration (MIC) and minimal bactericide concentration (MBC) (μL/mL) of TEO4, TEO5 and TEO6 on *S. mutans* ATCC700610. The TEOs were considered bactericidal when the MBC: MIC ratio was 2: 1, and bacteriostatic if this ratio was higher than 2: 1

**Fig. 1:**
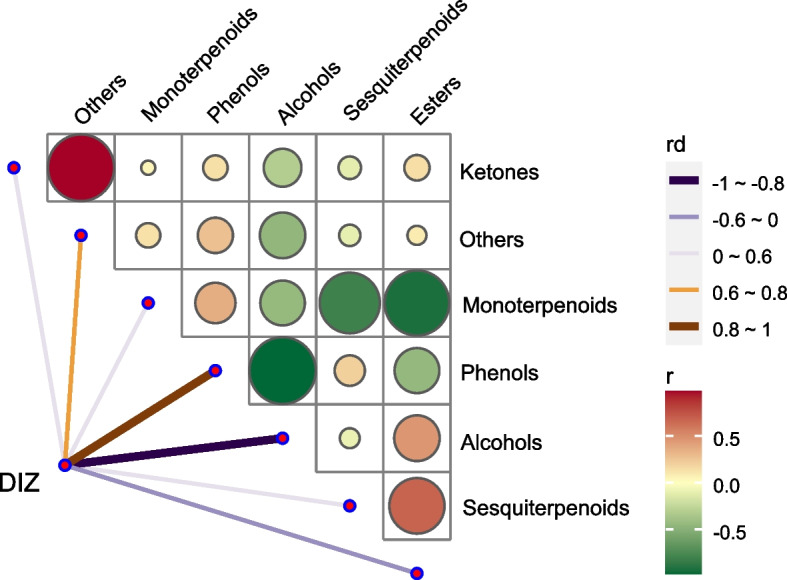
Correlation of TEOs components class with DIZ Values. Network between TEOs chemical Classes and DIZ. The Spearman correlation coefficients between genes and metabolites were calculated. The gene-metabolite pair shown in the network was chosen based on an adjusted p-value of 1E-5. The circles represent metabolites, whereas the triangles represent genes with a different hue for each co-expression module. The metabolites were colored according to their major classes. The edges were colored based on whether the correlation coefficient between genes and metabolites was positive or negative

As shown in Fig. 1, the Pearson correlation analysis of TEOs’ components and DIZ values showed that phenols and others might be the main constituents for inhibition of *S. mutans* growth, as phenols and others had positive connections with DIZ, the rd values were 0.849 and 0.692 respectively. The result also indicated that ketones and others had a positive connection (*r* = 0.973), followed by esters and sesquiterpenoids (r = 0.676) in TEOs. While the contents of monoterpenoids and esters, alcohols and phenols, monoterpenoids and sesquiterpenoids presented negative connections with r = -0.927, r = -0.975, and r = -0.805 respectively.

### MIC and MBC of TEOs

The MIC and MBC of each TEO against *S. mutans* were measured and the results were listed in Table 4. This result indicated that the inhibition of TEOs on *S. mutans* growth had a concentration-dependent tendency and varying extend. Among three TEOs, TEO5 showed stronger antibacterial activity with (MIC = 0.625 μL/mL; MBC = 1.25 μL/mL), followed by TEO6 (MIC = 0.625 μL/mL; MBC = 2.50 μL/mL), TEO4 was the last one with (MIC = 1.25 μL/mL; MBC = 5.0 μL/mL). The MBC:MIC ratio showed that most TEOs and control are bacteriostatic for *S. mutans*, except for TEO5. Among TEOs, only TEO5 considered bactericidal against *S. mutans*.

### Inhibition of acid production

**Fig. 2:**
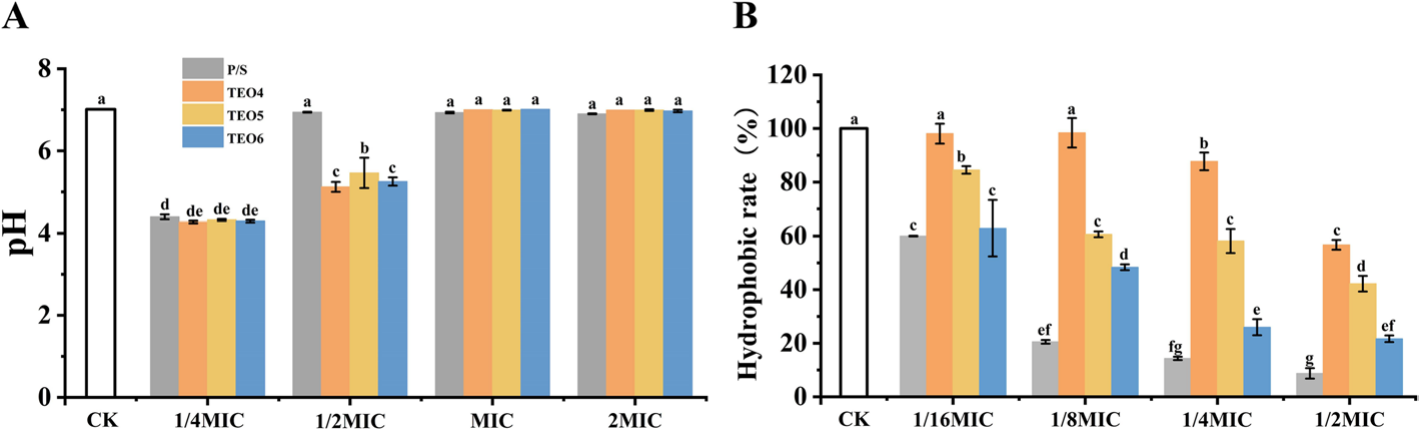
Effects of TEOs on the pH and hydrophobic rate of *S. mutans* ATCC700610 cultures. **A** pH; **B** hydrophobic rate. Values are means ± STD. Statistical analysis was performed by one-way ANOVA with a Waller-Duncan test. “a”; “b”; “c”; and “d” indicate significant differences (*p* < 0.05)

In order to decide whether three TEOs inhibit the acid production in *S. mutans*, the bacteria were cultured in the presence of various concentrations of the essential oil and the pH change was measured. As summarized in Fig. 2A, the pH was significantly decreased at control group (pH 4.17 ± 0.01). The pH decrease was significantly inhibited with MIC and 2MIC of TEOs treatments, and the inhibition levels were similar to the positive control. These results suggested that TEOs could inhibit the acid production ability of *S. mutans*, but they need proper dosage.

### Inhibition of bacteria hydrophobicity

The inhibition of TEOs on the surface hydrophobicity of *S. mutans* was determined by the microbial adhesion hydrocarbon of method (MATH). As shown in Fig. 2B, a certain concentration dependence of the inhibition was observed in all the three TEOs used. While in the meantime, most TEOs treatments could significantly download the surface hydrophobicity, especially for TEO5 and TEO6 (1/16—1/2MIC). At 1/2MIC, TEO6 has the greatest inhibition (21.66%) on the hydrophobicity of *S. mutans*, which followed by TEO5 (42.21%), and TEO4 (56.72%).

### Inhibitory effects of TEOs on biofilm formation

**Fig. 3:**
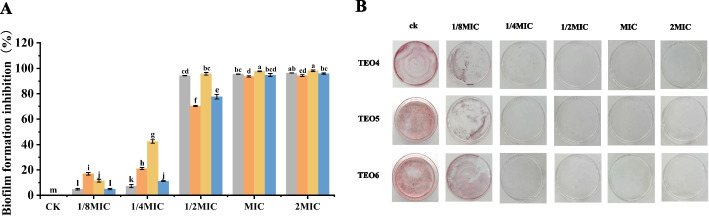
Effects of TEOs on biofilm formation inhibition of *S. mutans* ATCC700610. **A** crystal violet staining;** B** Safranine staining. Values are means ± STD. Statistical analysis was performed by one-way ANOVA with a Waller-Duncan test. “a”; “b”; “c”; and “d” indicate significant differences (*p* < 0.05)

As showed in Fig. 3, the biofilm formation of *S. mutans* was obviously inhibited by the three TEOs with a dose-dependent manner. Only the dosages were more than MIC, significant inhibition could be observed. Stronger anti-biofilm formation was found with TEO5 even at less dosage (< 1/2MIC). TEO5 reduced biofilm formation by 98.11% and 95.74% at 2MIC and 1/2MIC, while a similar or smaller effect was noted with TEO4 and TEO5, with the inhibitory rate by 94.31% and 70.31%, 95.90% and 77.60% respectively. In addition, the effect of the TEO5 on biofilm degradation was greater than the effect obtained with the positive control, regardless of the concentration used.

### Effects of TEOs on the mRNA expression of various virulence genes

**Fig. 4:**
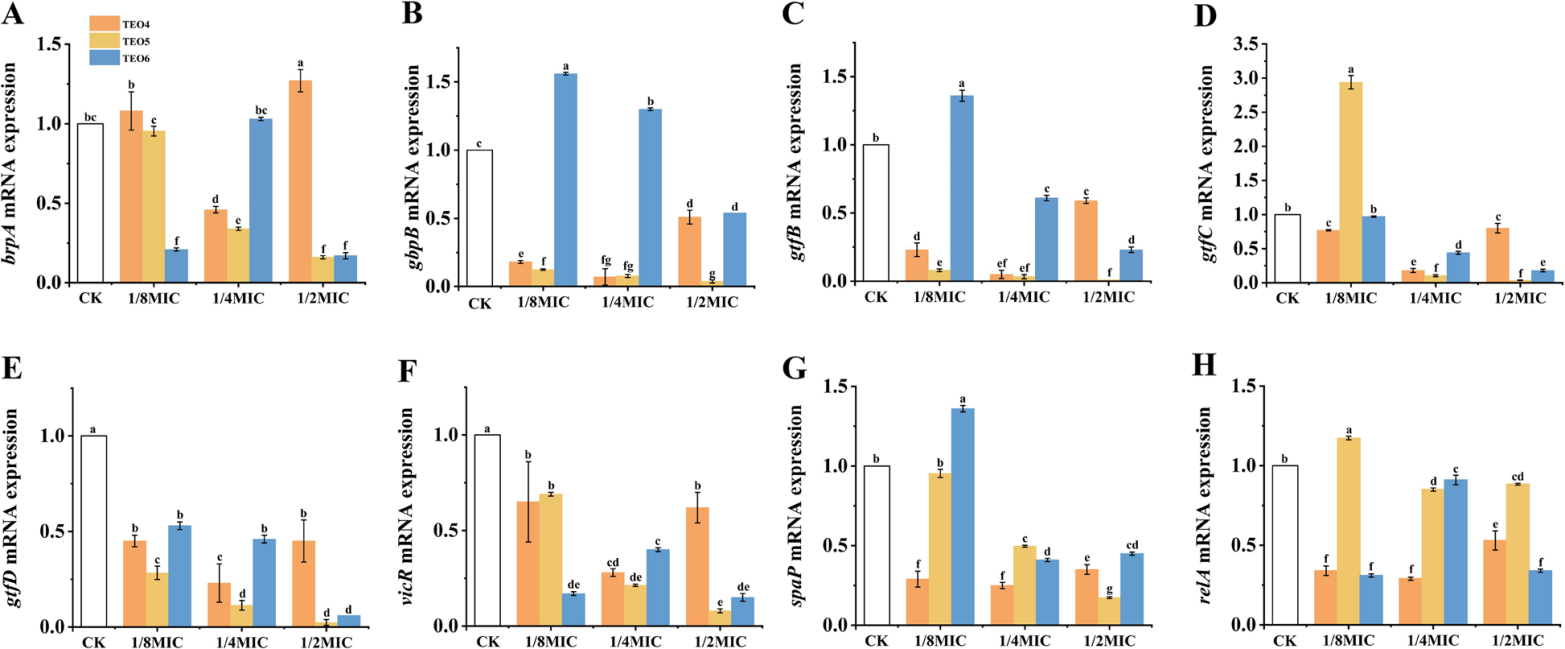
Effects of TEOs on genes expression of *S. mutans* ATCC700610. **A**: *brpA*; **B**: *gbpB*; **C**: *gtfB*; **D** *gtfC*; **E** *gtfD*; **F** *vicR*; **G** *spaP*; **H** *relA*. Values are means ± STD. Statistical analysis was performed by one-way ANOVA with a Waller-Duncan test. “a”; “b”; “c”; and “d” indicate significant differences (*p* < 0.05)

As shown in Fig. 4, in most cases expression of the *brpA*, *gbpB*, *gtfB*, *gtfC*, *gtfD*, *vicR*, *spaP* and *relA* decreased significantly (*p* < 0.05) in the presence of TEOs. At 1/2MIC TEOs concentration treatments, compared to the control all genes’ expression decreased more after TEO5 and TEO6 treatments. While the expression of *brpA* after TEO4 treatment was higher than control. The same phenomena were observed in some virulence genes’ mRNA expressions after lower TEOs concentrations treatments. Such as *gtfB*, *gbpB* and *spaP* expressed significantly higher than control following 1/8MIC TEO6 treatment, and the expression of *gtfC* and *relA* were significantly higher than control following 1/8MIC TEO5 treatment.

### Molecular docking

**Fig. 5:**
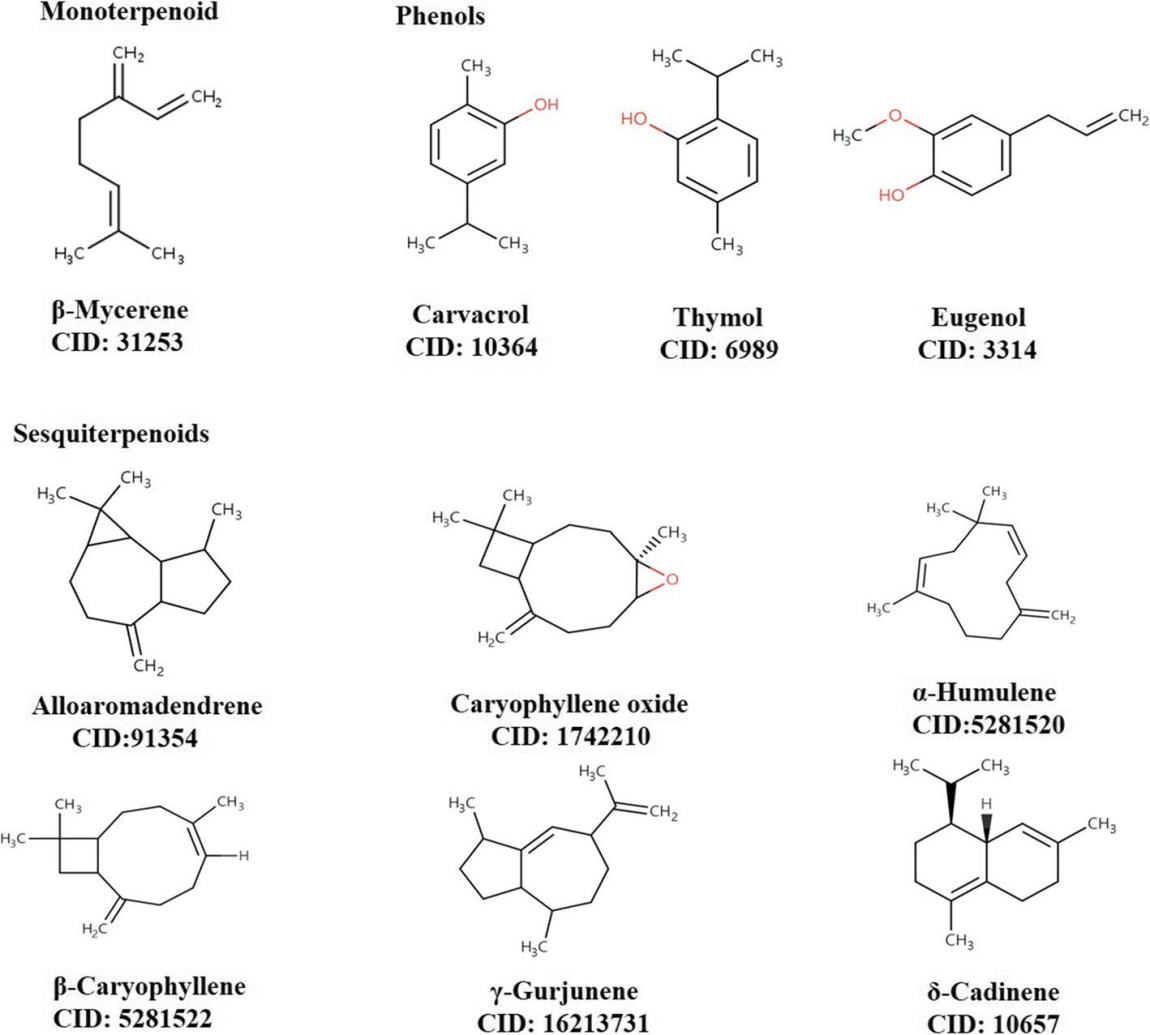
Compounds present in all TEOs with good concentration. One *Monoterpenoid*, three *Phenols* and six *Sesquiterpenoids* were selected as ligands based on GC–MS and Pearson correlation

**Fig. 6:**
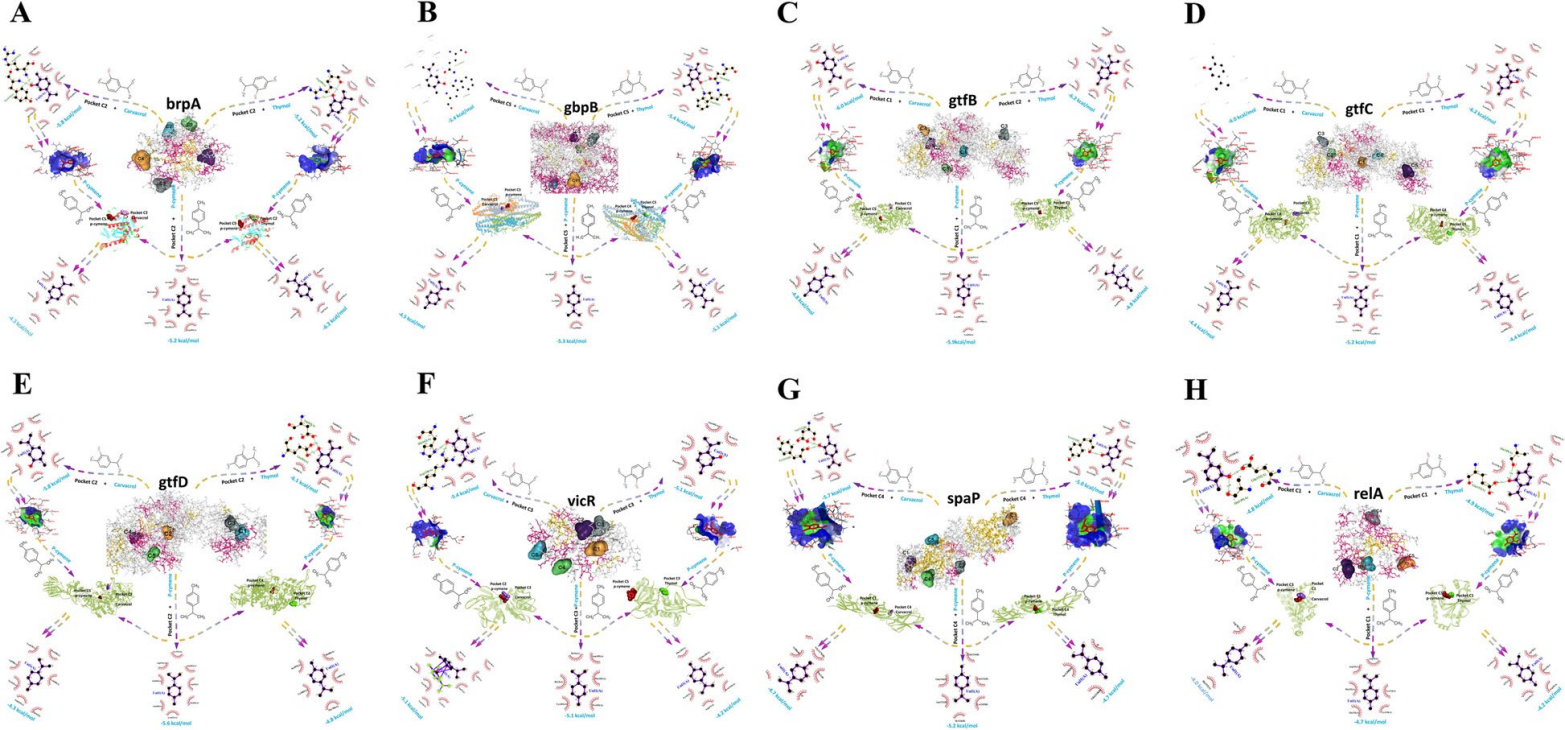
Molecular docking affinities and interactions of carvacrol, thymol, *p*-cymene individually and as complex with 8 virulent proteins of *S. mutans*. **A** *brpA* protein with ligands; **B** *gbpB* protein with ligands; **C** *gtfB* protein with ligands; **D** *gtfC* protein with ligands; **E** *gtfD* protein with ligands; **F** *vicR* protein with lligands; **G** *spaP* protein with ligands; **H** *relA* proteins with ligands. The colored arrows were presented according to binding affinity from best to comparatively less good (red < brown < green < blue < yellow)

From the Pearson correlation analysis of TEO components with DIZ and virulence gene expressions, 10 components were chosen based on the class and GC–MS percentages. The ADMET analysis of these proteins were presented in Table 5. The validation of designed proteins had been shown in Table S1 (see supplementary data). All the 10 components showed good interaction affinity (< -4.0) with the toxin proteins (Fig. 5). Among the proteins, *gbpB*, *gtfB*, *gtfC* and *gtfD* exerted more potentiality in interacting with all compounds as their affinities were comparatively the lowest (-7.6∽-5.4 kcal/mol). Carvacrol and thymol exhibited more consistent exchange of connection with the proteins as their affinity ranges from -6.2 kcal/mol to -4.8 kcal/mol (Fig. 6).

**Table 5:** ADMET analysis of 10 main components of *Thymus* essential oils

Compounds	Formula	MW (g/mol)	TPSA	Ali Solubility (mg/ml)	Ali Class	GI absorption	BBB permeant	Lipinski #violations	PAINS #alerts	Leadlikeness #violations
*β*-Myrcene	C_10_H_16_	136.23	0	1.80E-02	Soluble	Low	Yes	0	0	2
*γ*-Terpinene	C_10_H_16_	136.23	0	8.19E-03	Moderate	Low	Yes	0	0	2
*p*-Cymene	C_10_H_14_	134.22	0	2.10E-02	Soluble	Low	Yes	1	0	2
Linalool	C_10_H_18_O	154.25	20.23	1.35E-01	Soluble	High	Yes	0	0	1
Terpinen-4-ol	C_10_H_18_O	154.25	20.23	6.75E-02	Soluble	High	Yes	0	0	1
*α*-Terpineol	C10H18O	154.25	20.23	4.95E-02	Soluble	High	Yes	0	0	1
Eucalyptol	C_10_H_18_O	154.25	9.23	3.98E-01	Soluble	High	Yes	0	0	1
Carvacrol methyl ether	C_11_H_16_O	164.24	9.23	3.21E-02	Soluble	High	Yes	0	0	2
Carvacrol	C10H14O	150.22	20.23	3.79E-02	Soluble	High	Yes	0	0	1
Thymol	C10H14O	150.22	20.23	5.97E-02	Soluble	High	Yes	0	0	1

As presented in Tables 6 and S2-S4 (see supplementary data), the interacting residues of these two components with protein *brpA*, *gtfB* and *gtfD* were mostly bonded with covalent, pi and alkyl bonds while circled by van dar wales. Carvacrol had the lowest energy -5.8 kcal/mol to interact with the second pocket in *brpA* (C2-brpA) located on functional position (80-221AA), followed by *p*-cymene (-5.4 kcal/mol). Pockets in their catalytic domain Glyco hydro70cat were identified: pocket 1(C1) for *gtfB* and *gtfC* and pocket 2(C2) for *gtfD*. All of the components docked quite well with these three proteins, and the interaction connections were also very stable.

**Table 6:** Docking affinities and interactions of carvacrol and thymol with the virulence proteins

Proteins	Carvacrol			Thymol		
	**Binding Affinity** **(kcal/mol)**	**Interaction**		**Binding Affinity** **(kcal/mol)**	**Interaction**	
		**H/Alkyl/Covalent**	**Van der wales**		**H/Alkyl/Covalent**	**Van der wales**
***brpA***	-4.6	Ile 181, His 184, Arg 209, Tyr 212	Arg 103, Glu 185, Arg 211, Arg 221	-4.8	Ile 181, His 184, Arg 209, Tyr 212	Asp 82, Glu 185
***gbpB***	-6.8	Lys 96, Ala 99, Arg 100, Met 150	Ser 103	-6.3	Lys 96, Ala 99, Arg 100	Ser 103, Met 150, Gln 153
***spaP***	-5.6	Val 1256	Asp 1212, Tyr 1213, Pro 1214, Glu 1215, Glu 1216, Tyr 1309, Glu 1310	-5.3	Pro 1214, Lys 1265	Asp 1212, Tyr 1213, Glu 1216, Tyr 1309, Glu 1310
***relA***	-4.8	Tyr 114, His 153	Arg 43, Lys 45, Lys 53, Asp 70, Arg 75, Lys 110, His 118, Glu 137, Gln 139, Arg 141	-5.2	Tyr 114, Gln 139, His 153	Lys 53, Asp 70, Arg 75, Lys 110, Glu 137, Arg 141, Ala 149, Glu 152
***vicR***	-6.0	Ile 13, Ile 16, Ile 17, Lys 101, Pro 102, Phe 103	Asn 105	-4.9	Ile 13, Ile 16, Lys 101, Pro 102, Phe 103	Asn 105
***gtfB***	-7.5	Leu 407, Ala 452, Phe 881, Asp 890	Leu 408, Asp 451, Asn 455, Glu 489, Asp 562, Gln 566, Asp 883, Gln 934	-6.7	Leu 356, Leu 407, Trp 491, His 561, Tyr 584, Phe 881, Asp 890	Leu 408, Asp 451, Ala 452, Asn 455, Glu 489, Asp 562, Gln 566, Asp 567, Asp 883, Gln 934
***gtfC***	-7.5	Leu 382, Leu 433, Leu 434, Trp 517, Phe 907, Asp 909, Tyr 916	Asp 477, Ala 478, Asn 481, Glu 515, His 587, Asp 588, Gln 592, Gln 960	-6.6	Leu 382, Leu 433, Trp 517, Gln 592, Tyr 610, Phe 907, Tyr 916	Leu 434, Asp 477, Ala 478, Asn 481, Glu 515, Asp 588, Asp 909, Gln 960
***gtfD***	-7.1	Leu 373, Leu 421, Ala 466, His 583, Tyr 921	Leu 422, Asp 465, Glu 503, Asp 584, Gln 588, Asn 867, Asp 914, Phe 912, Asn 919	-6.7	Leu 373, Leu 421, Ala 466, His 583, Tyr 921	Leu 422, Asp 465, Asn 469, Glu 503, Trp 505, Asp 584, Gln 588, Phe 912, Asp 914

Carvacrol and thymol clearly interacted with *gbpB* protein sporting more stable bond with the interacting residues Lys 158, Val 161, Glu 162, Gln 165, Ala 323, Trp 352, Ala 365 inside the pocket 5. However, Eucalyptol docked with *gbpB* at the lowest energy but very close to carvacrol and thymol with -5.5 kcal/ mol. Pocket 4 of *spaP* (C4-spaP) were comprised of 1212-1312AA and interacted with carvacrol at smallest affinity (-5.7 kcal/mol). Thymol bonded with *relA* functional domain (pocket C1-relA: 53-153AA) at lowest -4.9 kcal/mol and terpene-4-ol at highest -3.5 kcal/mol affinity. The lowest affinity for *vicR* response regulator domain was -5.7 kcal/mol for eucalyptol and -5.6 kcal/mol for myrcene. All the docking results has been presented in Fig. S2-S9 (see supplementary data).

We predicted docking affinities with *p*-cymene, carvacrol-protein complex and thymol-protein complex (Table 7). The aim was to see the binding *p*-cymene at allosteric site. All the affinities of *p*-cymene at allosteric sites were impressive (above -4.0 kcal/mol). However, the best interaction of *p*-cymene was with the thymol-*brpA* complex with the lowest -6.2 kcal/mol affinity. The higher affinity was -4.0 kcal/mol for *p*-cymene and carvacrol-*relA* interaction. Overall, along with thymol and carvacrol, *p*-cymene, eucalyptol and myrcene showed the most interactions that could visibly influence the function of the proteins.

**Table 7:** Docking affinities and interactions of carvacrol-protein and thymol-protein complex with *p*-cymene

Complex	Binding Affinity (kcal/mol)	Interaction bonds
*brpA*-carvacrol	-4.3	Asp67 Thr68 Gly69 Glu72 Asn155 Met156 Glu157 Val160 Phe188 Glu202 Leu205 Asn255
*brpA*-thymol	-4.3	Asp67 Thr68 Gly69 Glu72 Asn155 Met156 Glu157 Val160 Phe188 Glu202 Leu205 Asn255
*spaP*-carvacrol	-4.7	Chain B: Glu1166 Gly1179 Ser1180 Thr1181 Tyr1183 Glu1378 Glu1379 Phe1381 Asn1480 Gly1481
*spaP*-thymol	-4.7	Chain B: Glu1166 Gly1179 Ser1180 Thr1181 Tyr1183 Glu1378 Glu1379 Phe1381 Asn1480 Gly1481
*relA*-carvacrol	-4.0	Chain A: Lys45 Lys53 Asp70 Lys110 Glu137 His153 Asn156
*relA*-thymol	-4.2	Chain A: Arg43 Lys45 Lys53 Asp70 Arg75 Arg103 Lys110 His118 Glu137 Gln139 His153
*vicR*-carvacrol	-5.1	Chain A: Pro46 Asp47 His72 Val73 Pro74 Leu116 Glu120 His162
*vicR*-thymol	-4.2	Chain A: Lys2 Asp47 His115 Leu116 Arg117 Arg118 Thr119 Glu120 Phe149 His162
*gtfB*-carvacrol	-4.8	Chain A: Asn599 Leu602 Leu603 Thr638 Lys647 Thr648 Ile649
*gtfB*-thymol	-4.8	Chain A: Asn599 Leu602 Leu603 Thr638 Lys647 Thr648 Ile649
*gtfC*-carvacrol	-4.4	Chain A: Val900 Ser901 Ser902 Thr919 Asp920 Asp923 Ser927 Pro965 Ser999 Asp1002
*gtfC*-thymol	-4.4	Chain A: Val900 Ser901 Ser902 Thr919 Asp920 Asp923 Ser927 Pro965 Ser999 Asp1002
*gtfD*-carvacrol	-4.3	Chain A: Thr536 Arg537 Pro538 Glu586 Val590 Lys629 Lys630 Tyr631 Thr632 Gln633
*gtfD*-thymol	-4.8	Chain A: Lys681 Ala682 Ile684 Lys685 Tyr686 Thr784 Asp831 Lys891 Ser892
*gbpB*-carvacrol	-4.5	Chain C: Ser318 Ala363 Ala364 Ala365 Gly366 Chain D: Ile97 Val98 Asn101 Gln154 Lys158 Gln396
*gbpB*-thymol	-5.1	Chain B: Ser95 Lys96 Ala99 Arg100 Ser103 Met150 Chain D: Lys96 Ala99 Arg100 Ser103 Met150

## Discussion

The genus *Thymus* is one of the most diverse and widespread plant families with antimicrobial properties [37]. The composition of TEOs varied between different *Thymus* species and varieties [38], and was affected by geographical location, geology, local climate and environmental conditions (temperature, sunshine, rainfall, etc.), acquisition time, nutrient content, plant genes, and methods of extracting EOs [39, 40]. The relative abundance of compositions in 6 TEOs tested in this study showed large variations, particularly in the amounts of carvacrol between 0 and 44.66%, thymol between 0 and 30.48%, *α*-terpineol between 0 and 46.09%, and linalool between 4.98 and 39.97%, *p*-cymene between 2.65 and 32.18%.

Our results are in partial agreement with those reported by Rota et al. [41] who studied the EO composition of *T. zygis* in Spain. The authors reported that thymol (68.1%) was the major component, followed by *p*-cymene (11.2%), *γ*-terpinene (4.8%) and carvacrol (3.5%). Ballester-Costa et al. [42] analyzed the EO composition of *T. vulgaris* collected from Serbia. The main components of the EO were linalool (44.7%) followed by terpineol-4 (11.8%),* γ*-terpinene (8.91%) and myrcene (6.89%) results that were in concordance with our study. On the contrary, Ramzi et al. [43] analyzed the EO composition of *T. satureioides* collected from Morocco. The main components of the EO were borneol as a major compound (48.0%), follow by *α*-terpineol (18.0%), camphene (14.4%), *α*-pinene (7.63%) and for some origins more or less contents of thymol (8.64%) or carvacrol (15.23%). These differences represent that the environmental conditions or genetic variations play an important role in the EO chemical variability.

The biological activity of the plant species is closely related to their chemical composition [44]. The major active components against *S. mutans* in TEOs were reported to be phenol compounds such as carvacrol and thymol [39]. Correlation analysis between relative abundance of components in TEOs and DIZ values revealed that the relative content of phenol was positively related to DIZ values. These results suggest that phenol components of TEOs are the potential active compounds. Because of their small size and lipophobic properties, EOs with a high phenol component could integrate into the bacterial cell membranes, easily cross lipid barriers and break the membrane structure, thereby disrupting cell growth and causing the cell death [17, 40]. Damtie et al. [25] reported that antibacterial effect in vitro of Ethiopian thyme species rich in thymol on *S. mutans*. The EO showed inhibitory activity on *S. mutans* with MIC and MBC values at 0.25, 0.5 μL/mL, respectively. On the contrary, the antimicrobial activity of EOs extracted from several *Thymus* species have been also reported against oral pathogens [19]. Result showed that high thymol content is not the only reason for antibacterial activity. Our results also indicated that the antibacterial effect is not due to the high concentration of phenol components, but to the synergistic effect between the very low concentrations of terpinene and *p*-cymene. Ultee et al. [45] reported that the combination of *p*-cymene and carvacrol may enhance the antibacterial activity of carvacrol, cause destabilization of the membrane and a decrease in the membrane potential.

Considering our GC–MS and antibacterial assay results, we take account of the multiple docking between *p*-cymene as well as carvacrol and thymol. Molecular docking is generally used to predict and inter molecular complex between the drug compounds with its target protein [46]. Our docking result showed that despite the presence of carvacrol or thymol in the functional sites, *p*-cymene could bind to the possible allosteric sites of the proteins. Based on the binding affinities, we hypothesize that *p*-cymene will inhibit protein activity if it enters the pocket containing the possible allosteric site. Even if it cannot bind to the site, carvacrol or thymol may be able to bind to the functional domain with low affinity and thus inhibit the protein. We hypothesize that TEO components can reduce *S. mutans* virulence in either case.

Moreover, TEOs could directly inhibit the acidogenicity, adhesion, and biofilm formation by *S. mutans*. RT-PCR results revealed that TEOs antibacterial properties are regulated by several genes encoding virulence factors. In *S. mutans*, *brpA*, *gbpB*, *gtfB*, *gtfC*, *gtfD*, *vicR*, *spaP* and *relA* work in a system to create virulence. So, we predicted our experimental TEO components as interacting molecules with the functional domains of these proteins. Each domain determines the functionality of the proteins. Enzymes function at varying rates depending on their surroundings. The environment's temperature, pH, location in the body, and the presence of other substances all have an effect on enzyme activity. Some substances bind to the enzyme in places other than the active site, which is referred to as the allosteric site. Molecules can use the allosteric site to activate, inhibit, or switch off enzyme activity. These molecules bind to the allosteric site and change the confirmation, or shape, of the enzyme. The biofilm regulating protein *brpA* has the principal functional domain LytR CpsA psr (79-236AA). This domain contains a short putative N-terminal cytoplasmic segment and a transmembrane segment that functions as a signal-anchor [47, 48]. *vicR* is a regulatory protein in *S. mutans* that aids in signal transduction. The response regulator receiver domain belongs to the CheY family. They receive the signal from the sensor partner in the two-component system, thus represented by the Sig transducer resp-reg receiver (2–116 AA) domain [49, 50]. This domain contained pocket 5 (C5-vicR) which was made up of 13-105AA. The catalytic enzymes *gtfB*, *gtfC*, and *gtfD* are primarily responsible for sucrose dependent adhesion by decomposing sucrose and generating biofilm [51]. They use sucrose to make glucans, and *gbpB* mediates their binding [52]. While pocket 1 and pocket 2 were the main targets for Gtfs, Pocket 5 (C5-gbp: 95-150AA) was identified in the Glucan-bd rpt (264–333 AA) functional domain of *gbpB* protein. This *gbpB* domain is the intrinsically disordered region which is considered ideal drug target for their active association with diseases. Adh_isopep-form_adh_dom (1155-1328AA) of *spaP* has a conserved Lys and Asn that form an intramolecular iso-peptide link. The domain can be found in a number of proteins, including cell-surface adhesins and Antigen I/II family members [53]. A pocket (C1-relA:53-153AA) was spotted in the HD/PDEase_dom of *relA* protein that is responsible for phosphohydrolase activity in *S. mutans* [54]. All 10 ligands exhibited impressive interaction to bind with the functional domain of virulent proteins. Among all the proteins, the interaction of *relA* and the components were comparatively less though the results of docking were promising.

Based on our in vitro and in silico findings, we theorize that if these components bind to the functional residues of these proteins and inhibit their activities, they could be useful for therapeutic alternatives to kill or inactivate *S. mutans*. Carvacrol and thymol seemed to be the most promising. These two components were not only more consistent in their connection than the others, but they were also more concentrated. Because all the components had the same molecular weight, it is possible that the highly concentrated components reacted with the proteins first, followed by the less concentrated ones. A thorough analysis and extensive research with pharmacokinetics are required to answer these questions.

## Conclusion

This study has proved that *Thymus* essential oils exhibited significant inhibition of bacterial growth, acid production, adherence, and biofilm formation of *S. mutans* depending on their constituents and relative concentration. These antimicrobial properties are regulated by several genes encoding virulence factors. According to GC-MS and molecular docking analysis, these activities are mainly attributed to the presence of the phenol compounds in their compositions. Our findings suggest that TEOs have the potential to be used for the prevention and treatment of the dental caries by *S. mutans*. Our results showed antibacterial activity of TEOs in vitro against *S. mutans*, but it should be borne in mind that the levels of EOs to inhibit bacterial growth in future dental product development are higher than in culture media. Due to this issue, further studies regarding pharmacokinetic and cytotoxicity are needed to evaluate the antibacterial activity in vivo and their clinical efficacy.

## Supplementary Information

**Additional file 1:** **Table S1.** Evaluation results of *brpA*, *relA*, *gtfB*, *gtfC*, *gtfD *3D structures. **Table S2.** Molecular Docking results with interacting residues of protein *brpA*, *gbpB*, *spaP* and compounds. **Table S3.** Molecular Docking results with interacting residues of protein *gtfB*, *gtfC*, *gtfD*, and compounds. Table S4 Molecular Docking results with interacting residues of protein *relA*, *vicR* and compounds. **Figure S1.** All 5 pockets detected in the 8 virulent proteins of *S.mutans. *Based on the sequence, the pockets in the functional domains of each protein were determined and presented with the name. **Figure S2.** Docking interaction of 8 components with* brpA* protein. A: the selected pocket in the functional domain of *brpA* protein; B: schematic presentation of docked complex interaction in 2D and 3D format. Blue to green range of surrounding depicts solubility of protein and different colors in 2D format represents different type of bonds. **Figure S3.** Docking interaction of 8 components with *gbpB* protein. A: the selected pocket in the functional domain of *gbpB* protein; B: schematic presentation of docked complex interaction in 2D and 3D format. Blue to green range of surrounding depicts solubility of protein and different colors in 2D format represents different type of bonds. **Figure S4.** Docking interaction of 8 components with *vicR* protein. A: the selected pocket in the functional domain of *vicR* protein; B: schematic presentation of docked complex interaction in 2D and 3D format. Blue to green range of surrounding depicts solubility of protein and different colors in 2D format represents different type of bonds. **Figure S5.** Docking interaction of 8 components with *gtfB *protein. A: the selected pocket in the functional domain of *gtfB *protein; B: schematic presentation of docked complex interaction in 2D and 3D format. Blue to green range of surrounding depicts solubility of protein and different colors in 2D format represents different type of bonds. **Figure S6.** Docking interaction of 8 components with *gtfC* protein. A: the selected pocket in the functional domain of *gtfC* protein; B: schematic presentation of docked complex interaction in 2D and 3D format. Blue to green range of surrounding depicts solubility of protein and different colors in 2D format represents different type of bonds. **Figure S7.** Docking interaction of 8 components with* gtfD* protein. A: the selected pocket in the functional domain of *gtfD* protein; B: schematic presentation of docked complex interaction in 2D and 3D format. Blue to green range of surrounding depicts solubility of protein and different colors in 2D format represents different type of bonds. **Figure S8**. Docking interaction of 8 components with* relA* protein. A: the selected pocket in the functional domain of* relA* protein; B: schematic presentation of docked complex interaction in 2D and 3D format. Blue to green range of surrounding depicts solubility of protein and different colors in 2D format represents different type of bonds. **Figure S9.** Docking interaction of 8 components with *spaP* protein. A: the selected pocket in the functional domain of* spaP *protein; B: schematic presentation of docked complex interaction in 2D and 3D format. Blue to green range of surrounding depicts solubility of protein and different colors in 2D format represents different type of bonds.

## Data Availability

The data used to support the findings of this study are available from the corresponding author upon request.

## Abbreviations

S. mutans: Streptococcus mutans
TEO: Thymus essential oil
GC–MS: Gas chromatography-mass spectrometry
BHI: Brain heart infusion
DIZ: Diameters of inhibition zones
MIC: Minimum inhibitory concentration
MBC: Minimum bactericidal concentration
P/S: Penicillin/streptomycin
