# Supplementary material for: Six Spain Thymus essential oils composition analysis and their in vitro and in silico study against Streptococcus mutans
Source: BMC Complement Med Ther. 2023 Apr 5;23:106. doi: 10.1186/s12906-023-03928-7 (PMC10074788; doi:10.1186/s12906-023-03928-7)
Supplement: Supplementary file 1 — Additional file 1: Table S1. Evaluation results of brpA, relA, gtfB, gtfC, gtfD 3D structures. Table S2. Molecular Docking results with interacting residues of protein brpA, gbpB, spaP and compounds. Table S3. Molecular Docking results with interacting residues of protein gtfB, gtfC, gtfD, and compounds. Table S4 Molecular Docking results with interacting residues of protein relA, vicR and compounds. Figure S1. All 5 pockets detected in the 8 virulent proteins of S.mutans. Based on the sequence, the pockets in the functional domains of each protein were determined and presented with the name. Figure S2. Docking interaction of 8 components with brpA protein. A: the selected pocket in the functional domain of brpA protein; B: schematic presentation of docked complex interaction in 2D and 3D format. Blue to green range of surrounding depicts solubility of protein and different colors in 2D format represents different type of bonds. Figure S3. Docking interaction of 8 components with gbpB protein. A: the selected pocket in the functional domain of gbpB protein; B: schematic presentation of docked complex interaction in 2D and 3D format. Blue to green range of surrounding depicts solubility of protein and different colors in 2D format represents different type of bonds. Figure S4. Docking interaction of 8 components with vicR protein. A: the selected pocket in the functional domain of vicR protein; B: schematic presentation of docked complex interaction in 2D and 3D format. Blue to green range of surrounding depicts solubility of protein and different colors in 2D format represents different type of bonds. Figure S5. Docking interaction of 8 components with gtfB protein. A: the selected pocket in the functional domain of gtfB protein; B: schematic presentation of docked complex interaction in 2D and 3D format. Blue to green range of surrounding depicts solubility of protein and different colors in 2D format represents different type of bonds. Figure S6. Docking interacti [file 12906_2023_3928_MOESM1_ESM.docx]

**Table S1** Evaluation results of *brpA*, *relA*, *gtfB*, *gtfC*, *gtfD* 3D structures

| **Protein name** | **Model** | **ERRAT** | **Verify 3D (%)** | **Favored region (%)** | **Allowed Region(%)** | **Non-allowed region(%)** |
| --- | --- | --- | --- | --- | --- | --- |
| ***brpA*** | I-tasser | 89.93 | 81.28 | 75.9 | 22.4 | 0 |
|  | Swiss-Model | 95.02 | 100 | 9.8 | 8.2 | 0 |
| ***relA*** | I-tasser | 99.53 | 80.54 | 82.3 | 14.8 | 1.5 |
|  | Swiss-Model | 97.33 | 76.12 | 96.8 | 3.2 | 0 |
| ***gtfB*** | I-tasser | 76.75 | - | 61.7 | 29.9 | 1.8 |
|  | Swiss-Model | 90.97 | 94.79 | 88.9 | 10.7 | 0 |
| ***gtfC*** | I-tasser | 76.87 | - | 64.6 | 27.8 | 3.2 |
|  | Swiss-Model | 92.05 | 94.91 | 89.8 | 10 | 0 |
| ***gtfD*** | I-tasser | 74.29 | - | 71.7 | 26 | 3.3 |
|  | Swiss-Model | 84.76 | 82.57 | 85.4 | 13.0 | 0.5 |

**Table S2** Molecular Docking results with interacting residues of protein *brpA*, *gbpB*, *spaP* and compounds

| **Compound** | ***brpA*** | | | ***gbpB*** | | | ***spaP*** | | |
| --- | --- | --- | --- | --- | --- | --- | --- | --- | --- |
|  | **Binding Affinity**  **(kcal/mol)** | **Interaction** | | **Binding Affinity**  **(kcal/mol)** | **Interaction** | | **Binding Affinity**  **(kcal/mol)** | **Interaction** | |
|  |  | **H/Alkyl/Covalent** | **Van der wales** |  | **H/Alkyl/Covalent** | **Van der wales** |  | **H/Alkyl/Covalent** | **Van der wales** |
| **Eucalyptol** | -4.1 | Ile 181, Arg 209, Arg 211, Tyr 212 | Asp 82, Arg 103, His 184 Glu 185, Arg 221 | -5.0 | Lys B:96, Lys D:96, Ala D:99, Arg D:100, Met D:150 | Ser D:103, Arg B:100, | -4.0 | Lys 1265 | Pro 1214, Glu 1216, Tyr 1309, Glu 1310 |
| ***γ*-Terpinene** | -5.3 | Ile 181, His 184, Tyr 212 | Ser 180, Glu 183, Glu 185, Arg 209, Met 210, Arg 211, Asp 213, Arg 221 | -5.1 | Lys B:96, Ala B:99, Ala D:99, Arg B:100, Arg D:100 | Lys D:96, Ser B:103, Ser B:103, Met B:150, Met D:150 | -4.0 | Lys 1265 | Asp 1212, Pro 1214, Glu 1216, Glu 1310, Asn 1311, Gln 1312, Ile 1326 |
| **Linalool** | -5.2 | Ile 181, His 184, Met 210, Arg 211, Tyr 212 | Ser 180, Glu 183, Glu 185, Arg 209, Asp 213, Arg 221 | -4.6 | Lys B:96, Lys D:96, Ala B:99, Ala D:99, Arg B:100, Arg D:100, Met B:150 | Ser D:103 | -3.7 | Pro 1214, Lys 1265 | Glu 1215, Glu 1216, Tyr 1309, Glu 1310 |
| ***β*-Myrcene** | -4.6 | Ile 181, His 184, Met 210, Arg 211, Tyr 212 | Arg 103, Ser 180, Glu 183, Glu 185, Arg 209, Arg 211, Arg 221 | -4.6 | Lys B:96, Lys D:96, Ala B:99, Ala D:99, Arg B:100, Arg D:100, Met B:150 | Ser B:103, Ser D:103 | -4.0 | Pro 1214, Lys 1265, Ile 1326 | Asp 1212, Tyr 1213, Glu 1216, Glu 1310, Asn 1311, Gln 1312 |
| ***p*-Cymene** | -5.4 | Ile 181, His 184, Tyr 212 | Ser 180, Glu 183, Glu 185, Arg 209, Met 210, Asp 213, Arg 221 | -5.2 | Lys B:96, Ala B:99, Ala D:99, Arg B:100, Arg D:100, | Lys D:96, Ser B:103, Ser D:103, Met B:150, Met D:150 | -5.2 | Lys 1265 | Asp 1212, Pro 1214, Glu 1216, Glu 1310, Asn 1311, Gln 1312, Ile 1326 |
| **Teerpinen-4-ol** | -5.1 | Ile 181, Glu 185, Arg 211, Tyr 212 | Arg 103, His 184, Arg 209, Arg 221 | -5.0 | Lys B:96, Lys D:96, Ala B:99, Ala D:99, Arg D:100, | Arg B:100, Ser B:103, Ser D:103, Met B:150 | -4.0 | Pro 1214, Lys 1265, Glu 1310 | Asp 1212, Tyr 1213, Glu 1216, Ser 1308, Tyr 1309 |
| ***α*-Terpinol** | -5.3 | Ile 181, His 184, Arg 211, Tyr 212 | Ser 180, Glu 183, Glu 185, Arg 209, Met 210, Arg 221 | -5.5 | Lys B:96, Lys D:96, Ala B:99, Ala D:99, Arg B:100, Arg D:100, Met B:150 | Ser B:103, Ser D:103, Met D:150 | -4.9 | Pro 1214, Glu 1216, Lys 1265, Ile 1326 | Asp 1212, Tyr 1213, Glu 1215, Glu 1310, Asn 1311, Gln 1312 |
| **Carvacrol-Methyl-Ether** | -4.7 | Ile 181, Glu 185, Arg 211, Tyr 212 | Arg 103, His 184, Arg 209, Met 210, Arg 221 | -5.2 | Lys B:96, Ala B:99, Ala D:99, Arg B:100, Arg D:100 | Lys D:96, Ser B:103, Ser B:103, Met B:150, Met D:150 | -5.0 | Lys 1265 | Asp 1212, Pro 1214, Glu 1216, Tyr 1309, Glu 1310, Asn 1311, Gln 1312, Ile 1326 |

**Table S3** Molecular Docking results with interacting residues of protein *gtfB*, *gtfC*, *gtfD*, and compounds

| **Compound** | ***gtfB*** | | | ***gtfC*** | | | ***gtfD*** | | |
| --- | --- | --- | --- | --- | --- | --- | --- | --- | --- |
|  | **Binding Affinity**  **(kcal/mol)** | **Interaction** | | **Binding Affinity**  **(kcal/mol)** | **Interaction** | | **Binding Affinity**  **(kcal/mol)** | **Interaction** | |
|  |  | **H/Alkyl/Covalent** | **Van der wales** |  | **H/Alkyl/Covalent** | **Van der wales** |  | **H/Alkyl/Covalent** | **Van der wales** |
| **Eucalyptol** | -4.1 | Leu 407, Leu 408, Ala 452, Phe 881, Tyr 890 | Leu 356, Asp 451, Glu 489, His 561, Asp 562, Gln 566, Asn 883, Phe 881, Asp 883, Gln 934 | -5.5 | Leu 433, Leu 434, Ala 478, Phe 907, Tyr 916 | Leu 382, Asp 477, Glu 515, His 587, Asp 588, Gln 592, Asp 909, Gln 960 | -5.1 | Leu 373, Leu 421, Leu 422, Ala 466, Phe 912, Tyr 921 | Arg 463, Asp 465, Glu 503, His 583, Asp 584, Gln 588, Asp 914, Gln 965 |
| ***γ*-Terpinene** | -5.3 | Leu 408, Tyr 890 | Leu 407, Asp 451, His 561, Gln 566, Phe 881, Asp 883, Gln 934 | -5.5 | Leu 434, Tyr 916 | Leu 433, Arg 475 Asp 477, His 587, Gln 592, Asp 909, Gln 960 | -6.0 | Leu 421, Leu 422, Ala 466, Trp 505, Tyr 921 | Leu 373, Asp 465, Asn 469, Glu 503, His 583, Asp 914, Gln 965 |
| **Linalool** | -5.2 | Leu 356, Leu 407, Leu 408, Asp 451, Ala 452, His 561, Tyr 584, Phe 881, Tyr 890 | Arg 449, Glu 489, Asp 562, Gln 566, Asp 883 | -4.6 | Leu 382, Leu 433, Leu 434, Ala 478, Asp 588, Tyr 610, Tyr 916 | Arg 475, Asp 477, Glu 515, His 587, Gln 592, Asp 909 | -5.0 | Leu 373, Leu 421, Leu 422, Ala 466, His 583, Asp 584, Tyr 921 | Arg 463, Asp 465, Glu 503, Gln 588, Phe 912, Asp 914, Gln 965 |
| ***β*-Myrcene** | -4.6 | Leu 356, Leu 407, Leu 408, His 561, Phe 881, Tyr 890 | Arg 449, Asp 451, Asp 562, Gln 566, Asp 883, Asn 836, Asn 888, Gln 934 | -5.0 | Leu 382, Leu 433, Leu 434, His 587, Phe 907, Tyr 916 | Arg 475, Asp 477, Asp 588, Gln 592, Asn 862, Asp 909, Asn 914, Gln 960 | -4.7 | Leu 373, Leu 421, Leu 422, His 583, Tyr 921 | Arg 463, Asp 465, Ala466, Glu 503, Asp 584, Gln 588, Phe 912, Asp 914 |
| ***p*-Cymene** | -5.4 | Leu 407, Tyr 890 | Arg449, Asp 451, His 561, Gln 566, Asp 883, Gln 934 | -5.3 | Leu 434, Tyr 916 | Leu 433, Arg 475, His 587, Gln 592, Asp 909, Gln 960 | -5.6 | Leu 421, Ala 466, Trp 505, Tyr 921 | Leu 373, Leu 422, Asp 465, Asn 469, Glu 503, His 583, Asp 914, Gln 965 |
| **Teerpinen-4-ol** | -5.1 | Leu 356, Tyr 584, Tyr 890 | Leu 407, Leu 408, Arg 449, Asp 451, Asp 451, Asp 562, Gln 566, Asn 836, Phe 881, Asp 883, Gln 934 | -4.6 | Leu 382, Tyr 610, Asp 909, Tyr 916 | Leu 433, Leu 434, Arg 475, Asp 477, His 587, Asp 588, Gln 592, Phe 907, Asn 914 | -5.1 | Leu 421, Asp 465, Trp 505, His 583, Tyr 921 | Arg 463, Glu 503, Asp 584, Asp 914 |
| ***α*-Terpinol** | -5.3 | Leu 356, Leu 407, Tyr 890 | Leu 408, Arg 449, Asp 451, Ala 452, Gln 489, His 561, Gln 566, Phe 881, Asp883, Gln 934 | -5.1 | Leu 382, Leu 433, Asp 477, His 587, Tyr 610, Phe 907, Tyr 916 | Leu 434, Ala 478, Gln 592, Asp 909, Gln 960 | -5.5 | Leu 421, Leu 422, Tyr 921 | Arg 463, Asp 465, Ala466, Glu 503, His 583, Gln 588, Phe 912, Asp 914, Gln 965 |
| **Carvacrol-methyl-ether** | -5.8 | Leu 356, Leu 407, Glu 489, Tyr 890 | Leu 408, Asp 451, Asn 455, Trp 491, His 561, Gln 566, Phe 881, Asp 883, Gln 934 | -5.8 | Leu 382, Leu 433, Gln 592, Tyr 610, Phe 907, Asp 909, Tyr 916 | Leu 434, Arg 475, Asp 477, His 587, Asp 588, Gln 960 | -5.4 | Leu 373, Leu 421, Ala 466, Asn 469, Glu 503, Trp 505, Tyr 921 | Leu 422, Asp 465, His 583, Phe 912, Asp 914, Gln 965 |

**Table S4** Molecular Docking results with interacting residues of protein *relA*, *vicR* and compounds

| **Compound** | ***relA*** | | | ***vicR*** | | |
| --- | --- | --- | --- | --- | --- | --- |
|  | **Binding Affinity**  **(kcal/mol)** | **Interaction** | | **Binding Affinity**  **(kcal/mol)** | **Interaction** | |
|  |  | **H/Alkyl/Covalent** | **Van der wales** |  | **H/Alkyl/Covalent** | **Van der wales** |
| **Eucalyptol** | -5.3 | Lys 110, Tyr 114, Ala 149, His 153 | Lys 45, Lys 53, Arg 75, Gln 139, Arg 141, Glu 152 | -7.0 | Lys 96, Ala 99, Arg 100, Met 150 | Ser 103 |
| ***γ*-Terpinene** | -5.0 | Tyr 114, His 153 | Lys 110, Ser 112, Arg 141, Ala 149, Glu 152, Asn 156 | -6.8 | Lys 96, Ala 99, Arg 100, Met 150 | Ser 103 |
| **Linalool** | -5.2 | Tyr 114, His 118, Ala 149, His 153 | Lys 53, Arg 75, Lys 110, Gln 139, Arg 141, Thr 150, Glu 152 | -6.9 | Lys 96, Ala 99, Arg 100, Met 150 | Ser 103, Met 150 |
| ***β*-Myrcene** | -5.2 | Tyr 114, His 118, Gln 139, Arg 141, His 153 | Arg 43, Lys 45, Lys 53, Arg 75, Arg 103, Lys 110, Glu 137 | -6.8 | Lys 96, Ala 99, Arg 100 | Arg 100, Ser 103, Met 150, Gln 153, Asp 157 |
| ***p*-Cymene** | -5.0 | Arg 75, Lys 110, Tyr 114, Arg 141, His 153 | Ser 112, Glu 137, Ala 149, Thr 150, Glu 152 | -6.6 | Lys 96, Ala 99, Arg 100 | Ala 99, Ser 103, Met 150, Gln 153 |
| **Teerpinen-4-ol** | -4.8 | Tyr 114, Glu 152, His 153 | Arg 75, Lys 110, Ser 112, Arg 141, Gln 139, Ala 149 | -6.3 | Lys 96, Ala 99, Arg 100 | Ser 103, Met 150, Gln 153 |
| ***α*-Terpinol** | -5.3 | Lys 110, Tyr 114, Ala 149, His 153 | Lys 45, Lys 53, Arg 75, Gln 139, Arg 141, Glu 152 | -5.4 | Lys 158, Val 161, Glu 162, Gln 165, Ala 323, Trp 352, Ala 365 | Asn 325, Gly 349, Asn 350, Tyr 351, Ser 361, Ala 362, Tyr 367 |
| **Carvacrol-Methyl-ether** | -4.7 | Lys 110, Try 114, Gln 139, His 153 | Ser 112, Gln 139, Arg 141, Ala 149, Glu 152 | -5.4 | Ile 13, Ile 16, Lys 101, Pro 102, Phe 103 | Ile 17, Asn 105 |


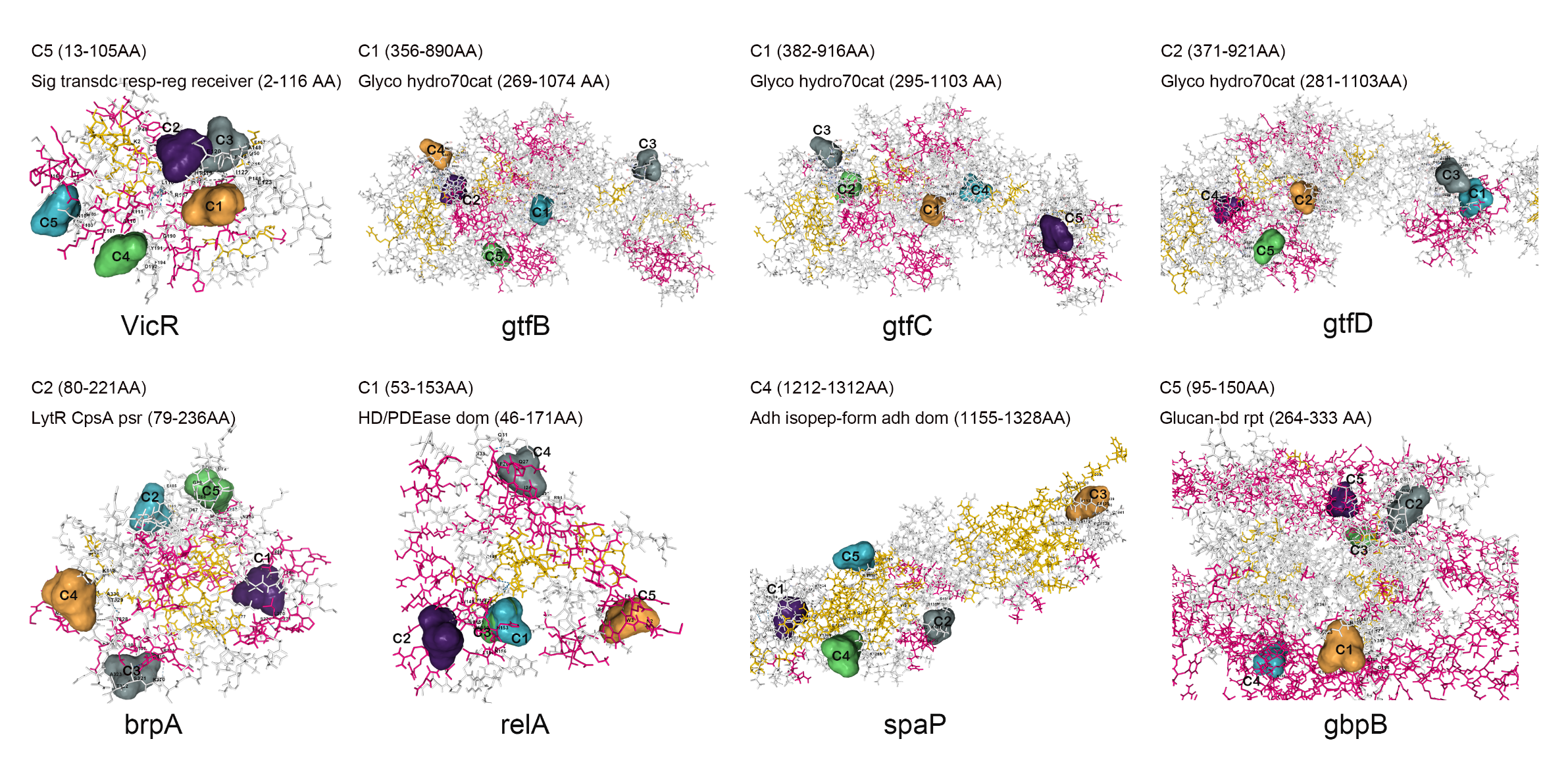


**Fig. S1** All 5 pockets detected in the 8 virulent proteins of *S.mutans.* Based on the sequence, the pockets in the functional domains of each protein were determined and presented with the name.


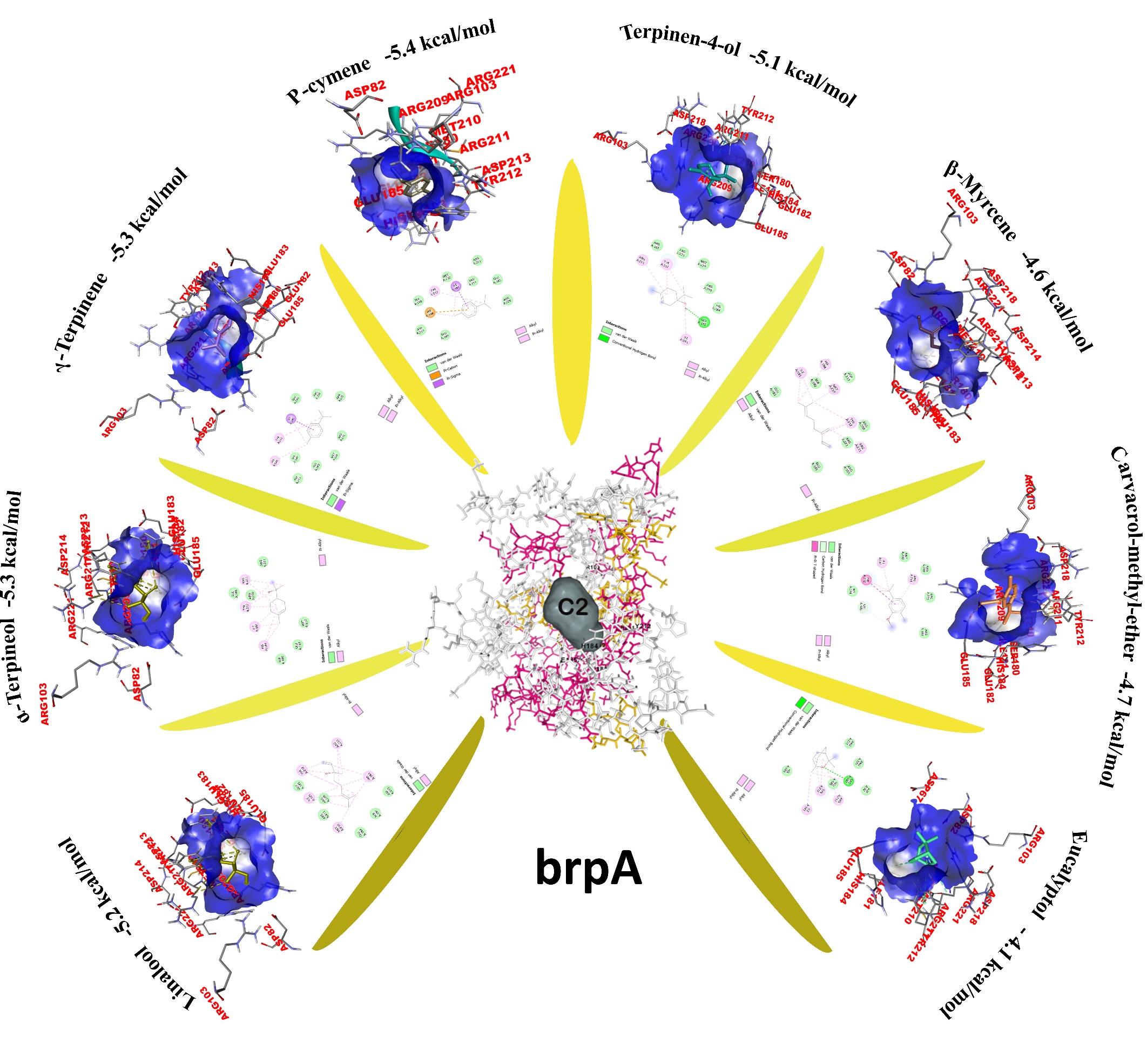


**A**

**B**

**Fig. S2** Docking interaction of 8 components with *brpA* protein. **A:** the selected pocket in the functional domain of *brpA* protein; **B:** schematic presentation of docked complex interaction in 2D and 3D format. Blue to green range of surrounding depicts solubility of protein and different colors in 2D format represents different type of bonds.


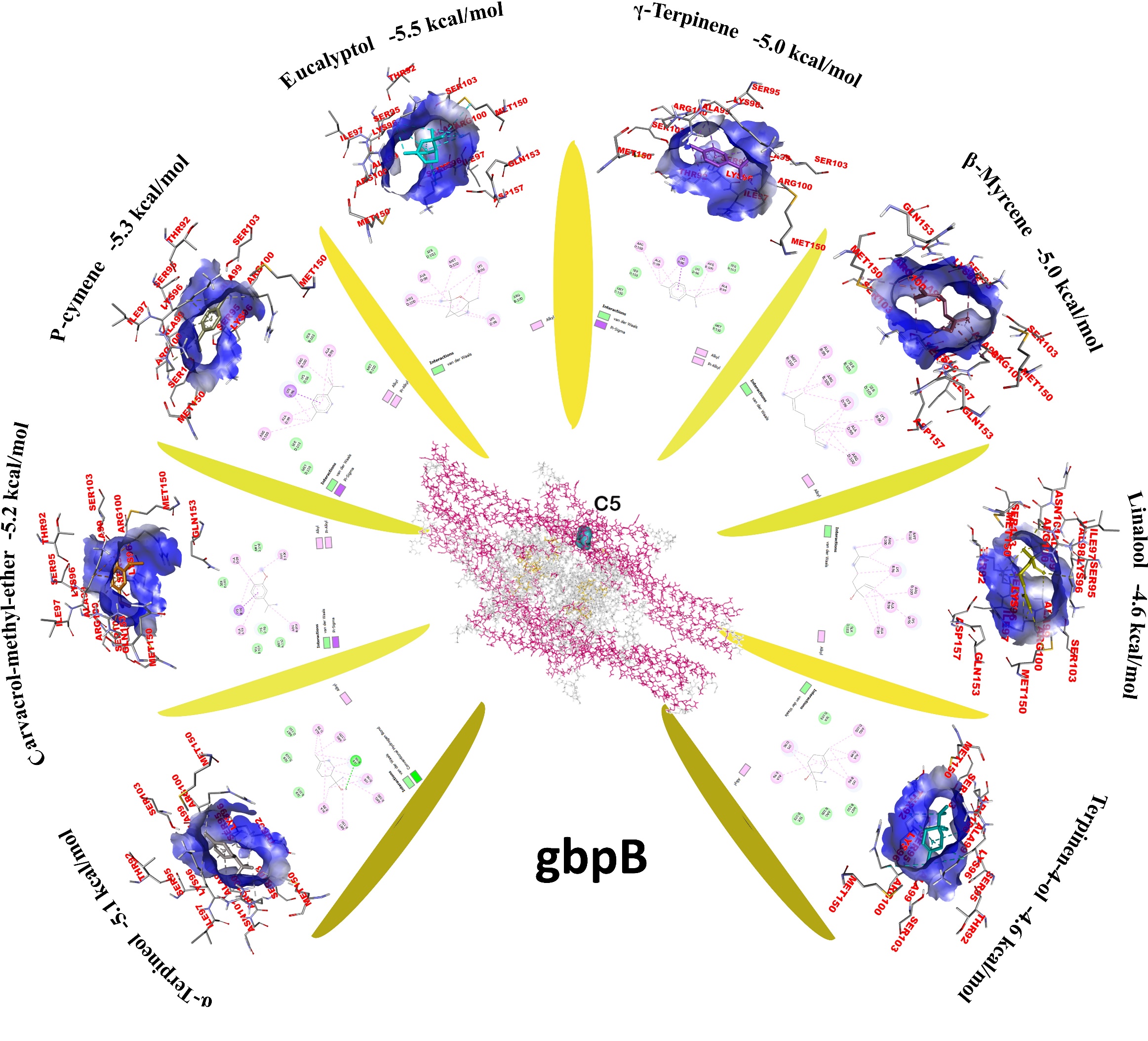


**B**

**A**

**Fig. S3** Docking interaction of 8 components with *gbpB* protein. **A:** the selected pocket in the functional domain of *gbpB* protein; **B:** schematic presentation of docked complex interaction in 2D and 3D format. Blue to green range of surrounding depicts solubility of protein and different colors in 2D format represents different type of bonds.


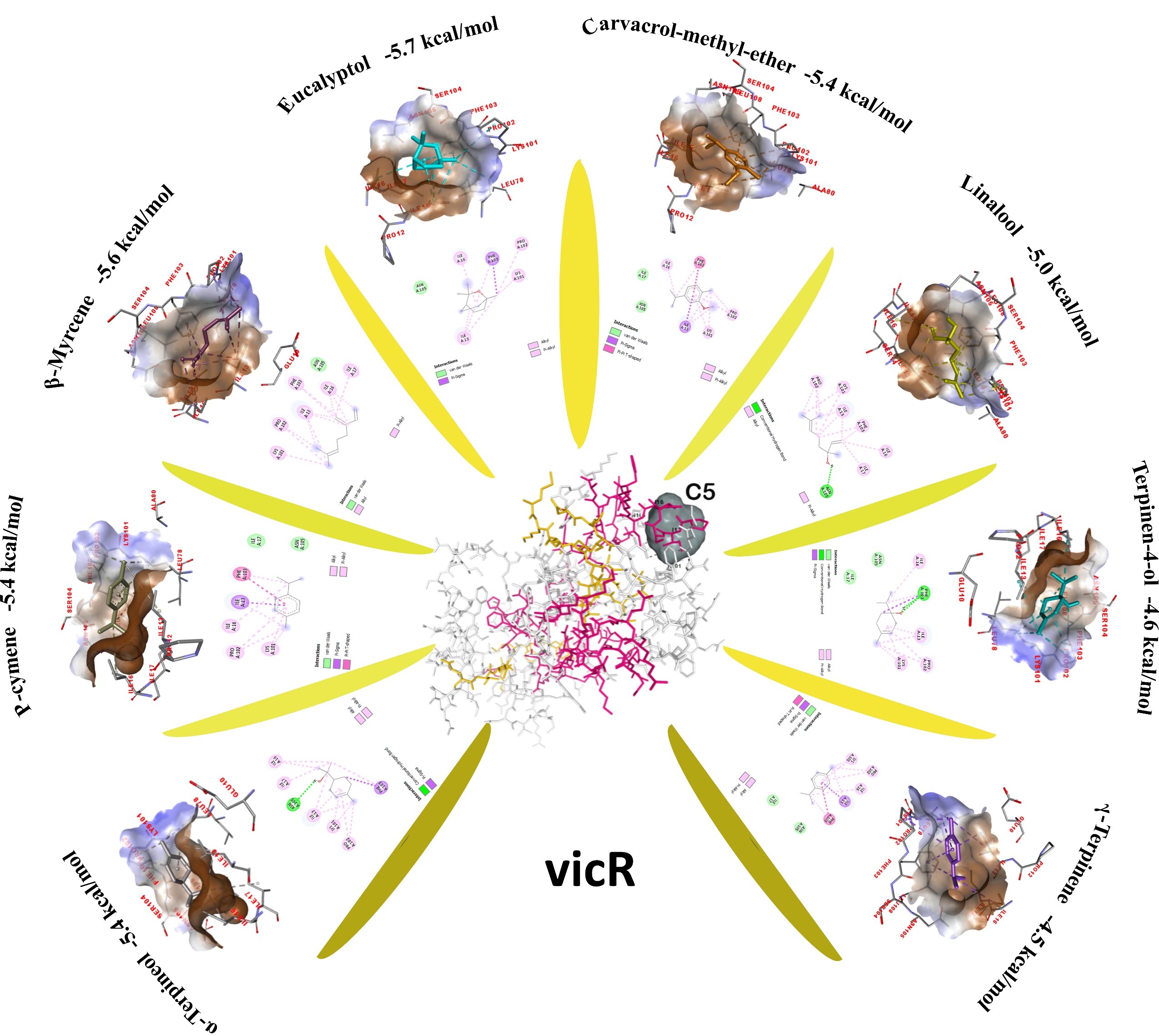


**B**

**A**

**Fig. S4** Docking interaction of 8 components with *vicR* protein. **A:** the selected pocket in the functional domain of *vicR* protein; **B:** schematic presentation of docked complex interaction in 2D and 3D format. Blue to green range of surrounding depicts solubility of protein and different colors in 2D format represents different type of bonds.


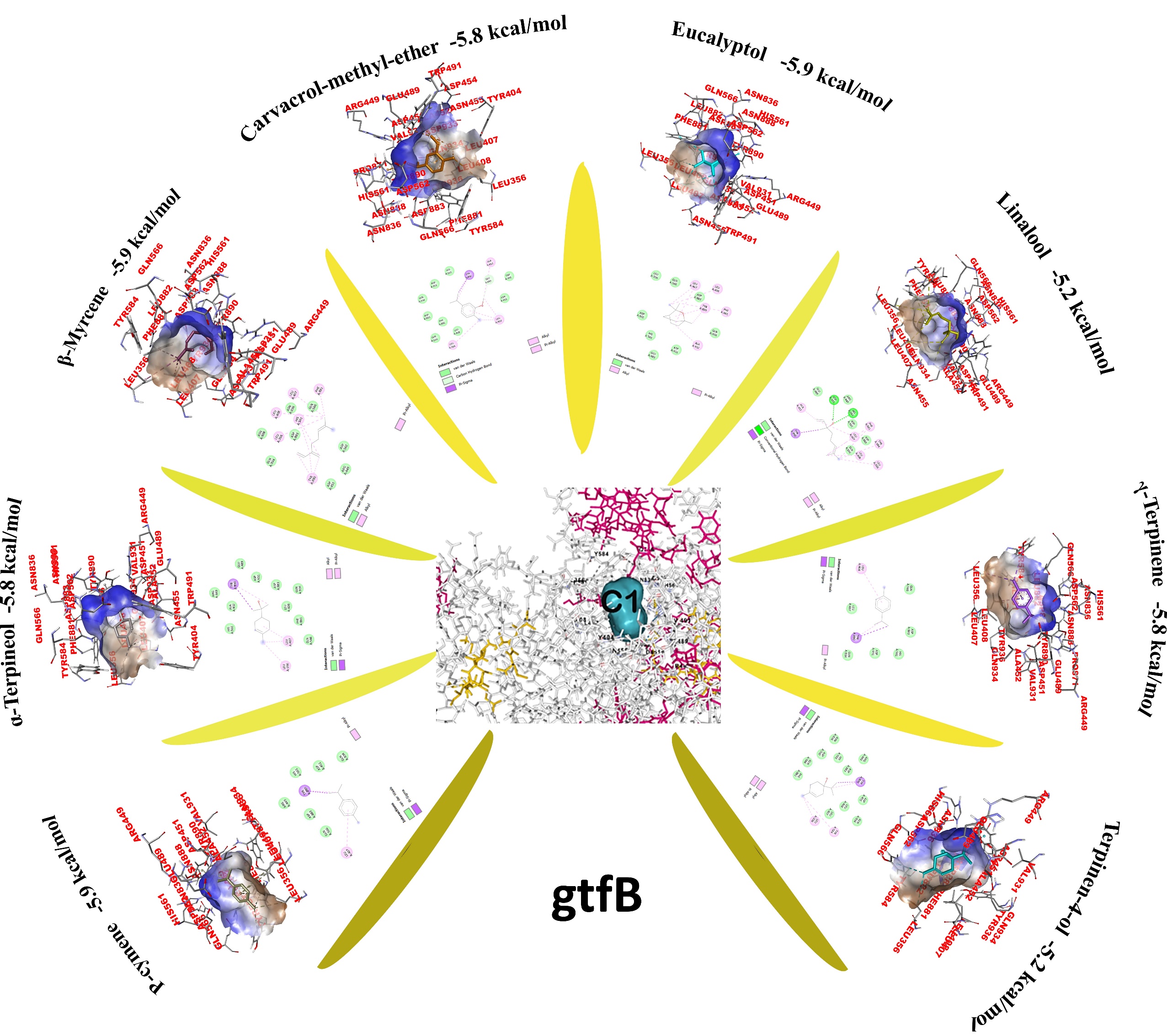


**B**

**A**

**Fig. S5** Docking interaction of 8 components with *gtfB* protein. **A:** the selected pocket in the functional domain of *gtfB* protein; **B:** schematic presentation of docked complex interaction in 2D and 3D format. Blue to green range of surrounding depicts solubility of protein and different colors in 2D format represents different type of bonds.


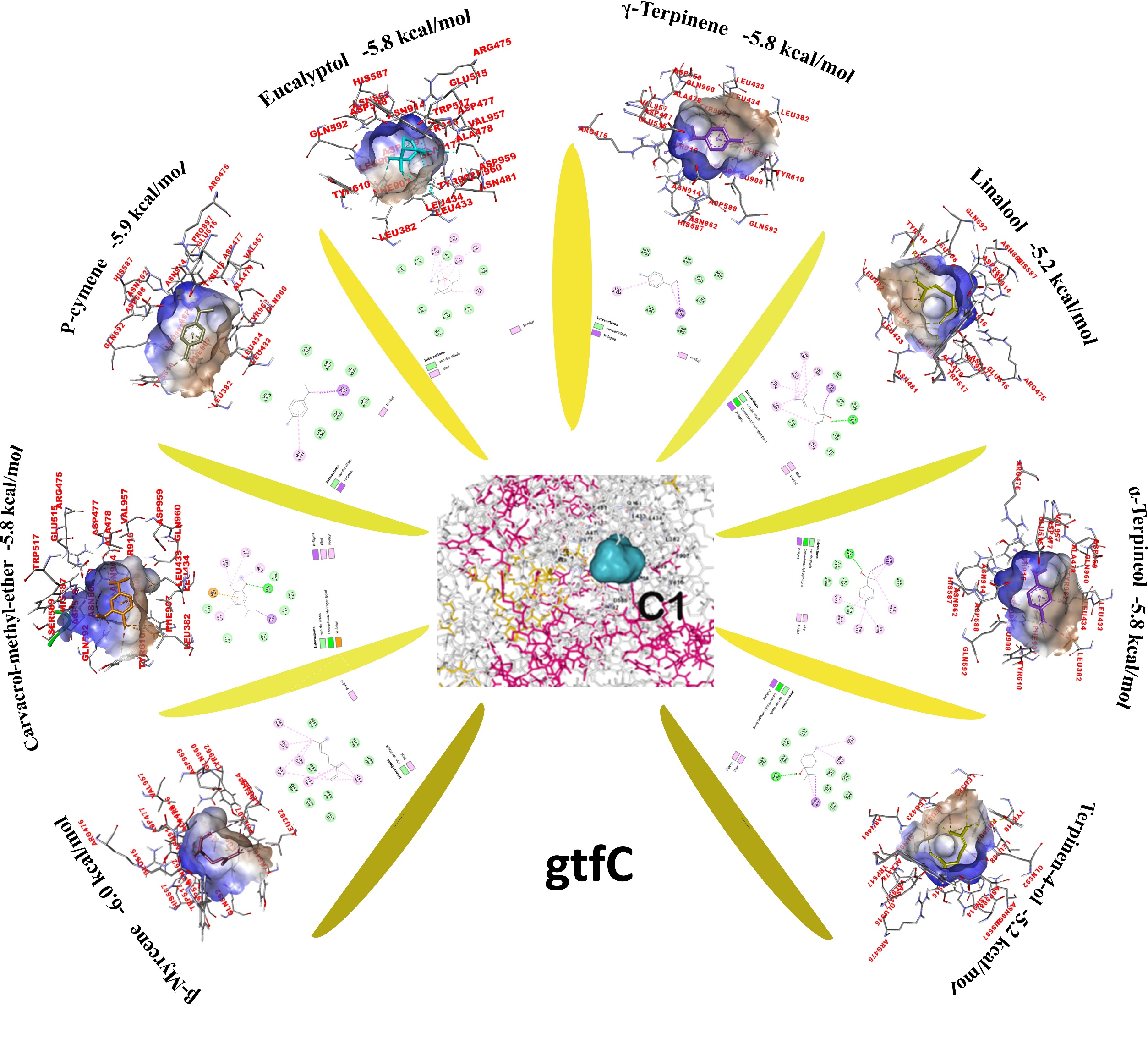


**B**

**A**

**Fig. S6** Docking interaction of 8 components with *gtfC* protein. **A:** the selected pocket in the functional domain of *gtfC* protein; **B:** schematic presentation of docked complex interaction in 2D and 3D format. Blue to green range of surrounding depicts solubility of protein and different colors in 2D format represents different type of bonds.


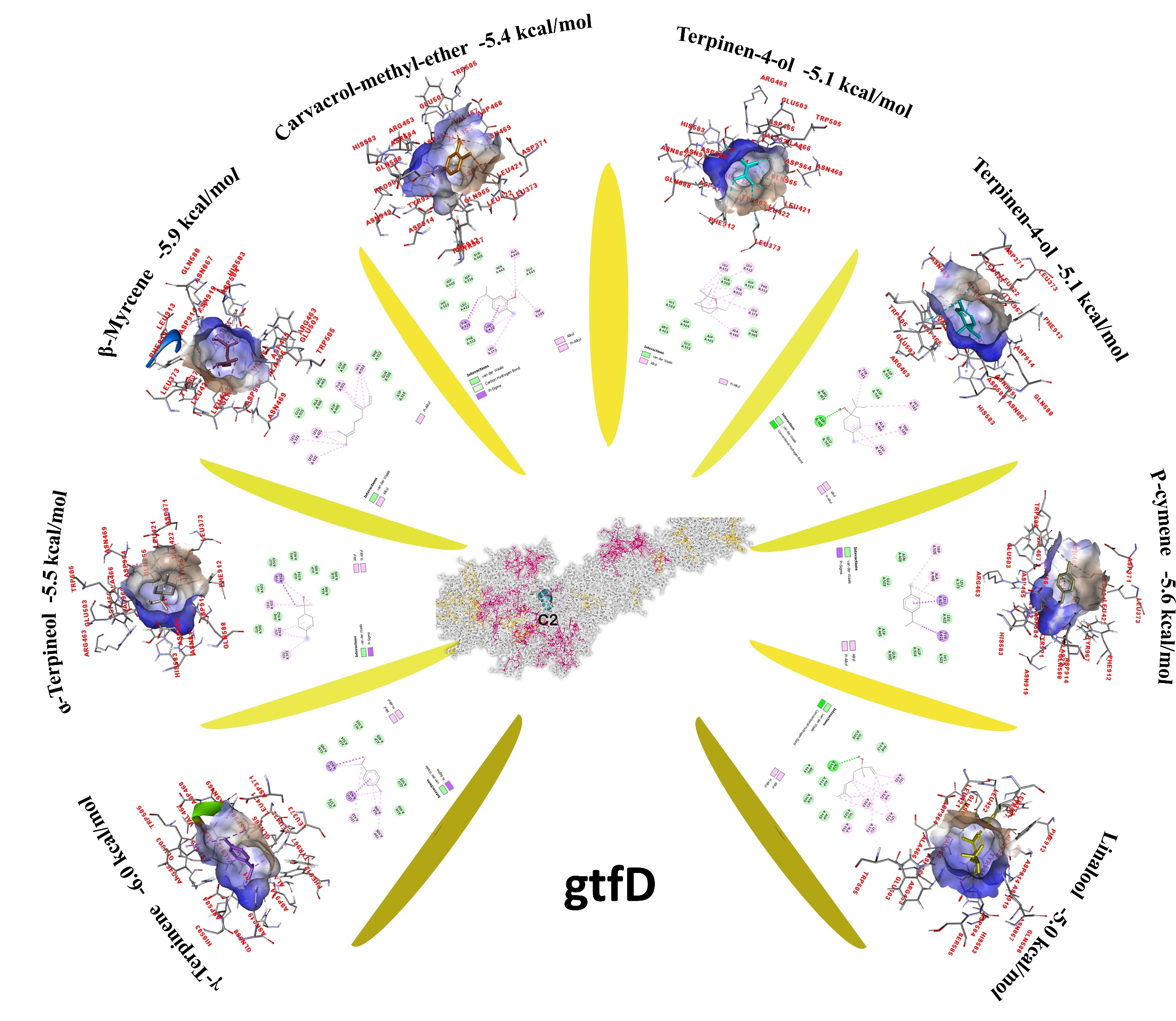


**B**

**A**

**Fig. S7** Docking interaction of 8 components with *gtfD* protein. **A:** the selected pocket in the functional domain of *gtfD* protein; **B:** schematic presentation of docked complex interaction in 2D and 3D format. Blue to green range of surrounding depicts solubility of protein and different colors in 2D format represents different type of bonds.


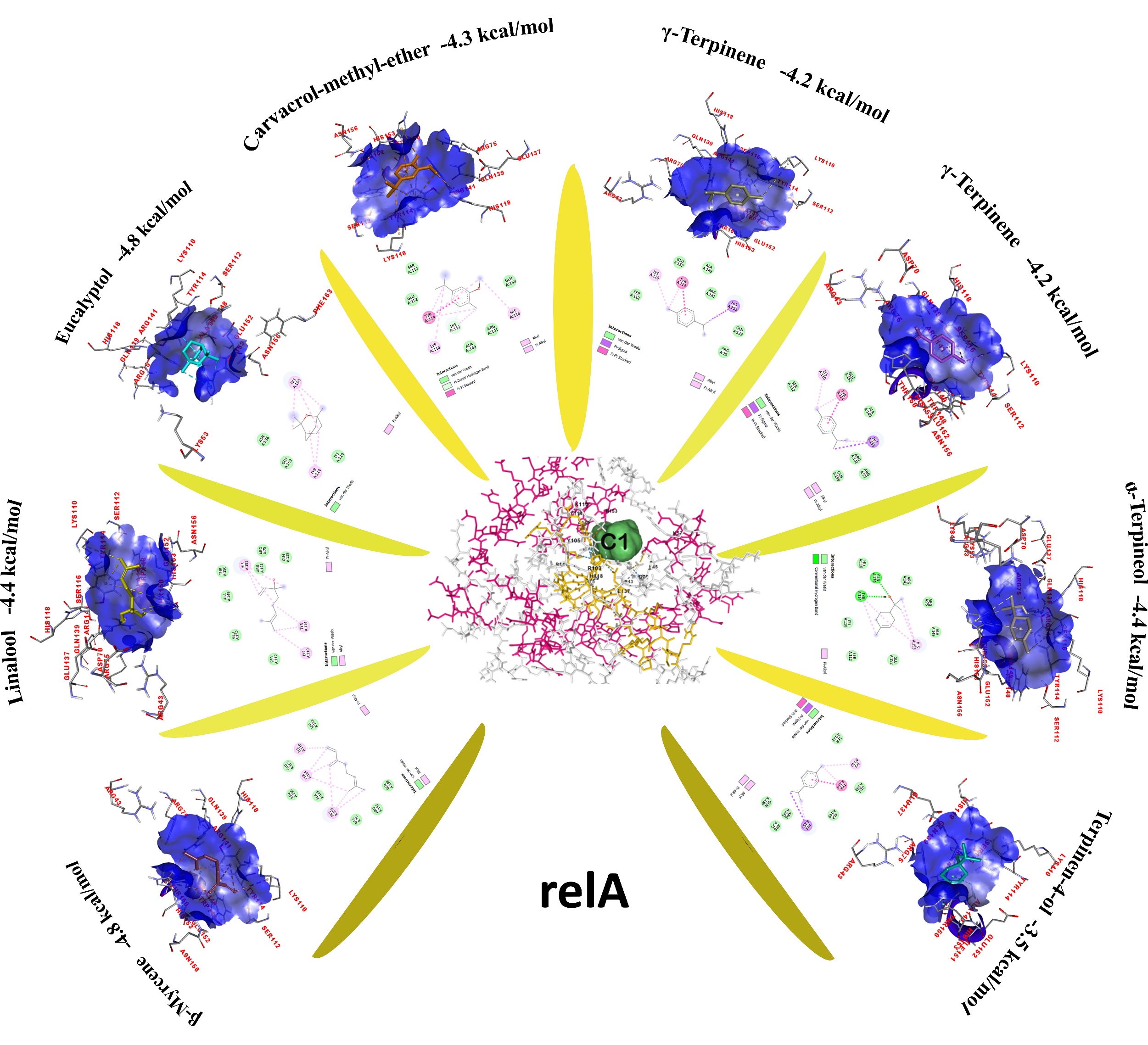


**B**

**A**

**Fig. S8** Docking interaction of 8 components with *relA* protein. **A:** the selected pocket in the functional domain of *relA* protein; **B:** schematic presentation of docked complex interaction in 2D and 3D format. Blue to green range of surrounding depicts solubility of protein and different colors in 2D format represents different type of bonds.


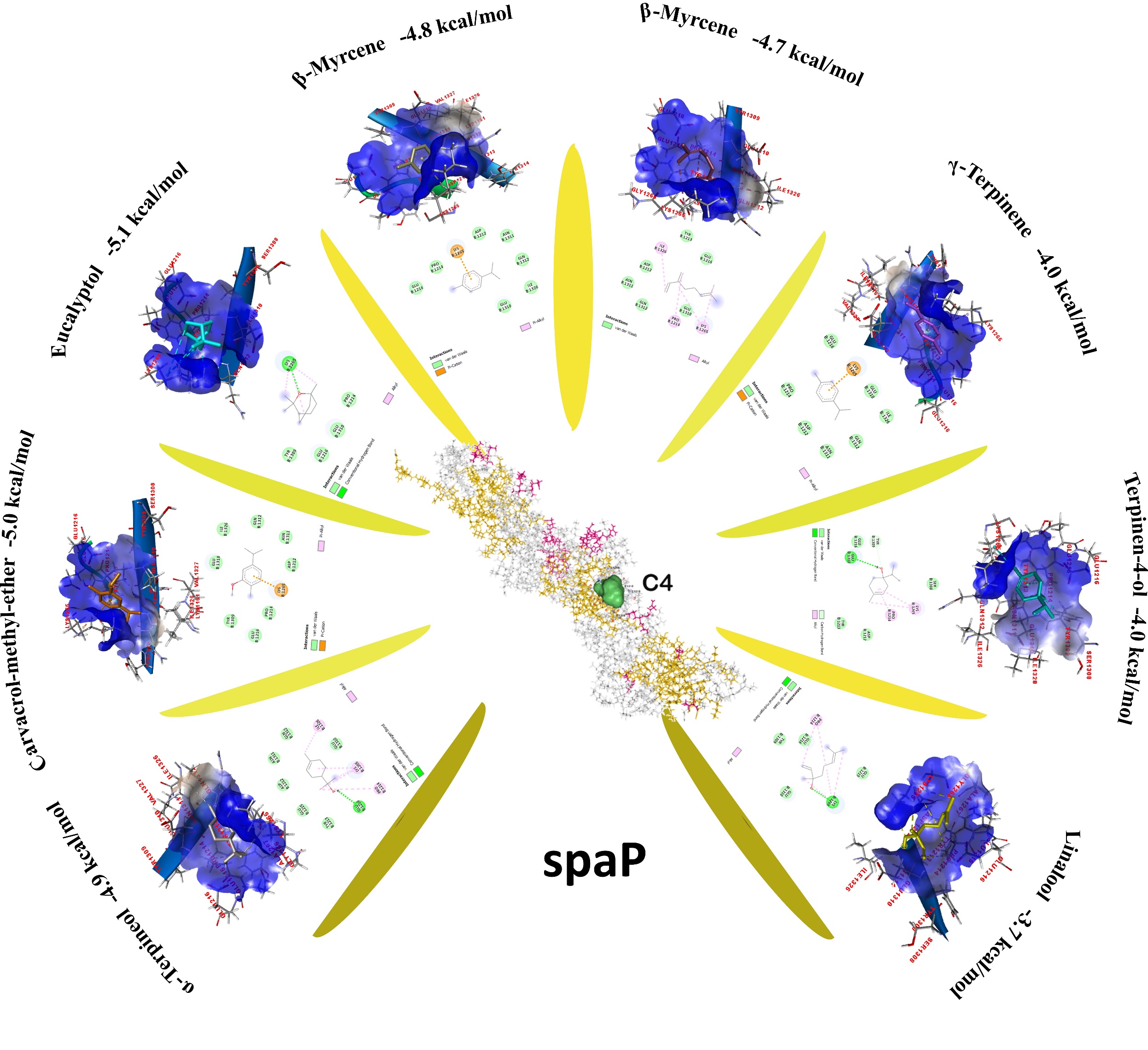


**B**

**A**

**Fig. S9** Docking interaction of 8 components with *spaP* protein. **A:** the selected pocket in the functional domain of *spaP* protein; **B:** schematic presentation of docked complex interaction in 2D and 3D format. Blue to green range of surrounding depicts solubility of protein and different colors in 2D format represents different type of bonds.
